# The ABC of happiness: Validation of the tridimensional model of subjective well-being (affect, cognition, and behavior) using Bifactor Polytomous Multidimensional Item Response Theory

**DOI:** 10.1016/j.heliyon.2024.e24386

**Published:** 2024-01-12

**Authors:** Ali Al Nima, Danilo Garcia, Sverker Sikström, Kevin M. Cloninger

**Affiliations:** a Department of Psychology, University of Gothenburg, Gothenburg, Sweden; b Promotion of Health and Innovation Lab (PHI), International Network for Well-Being, Sweden; c Department of Behavioral Sciences and Learning, Linköping University, Linköping, Sweden; d Centre for Ethics, Law and Mental Health (CELAM), University of Gothenburg, Gothenburg, Sweden; e Department of Psychology, Lund University, Lund, Sweden; f Anthropedia Foundation, St. Louis, Missouri, USA; g Promotion of Health and Innovation Lab (PHI), International Network for Well-Being, USA

**Keywords:** ABC of happiness, Subjective well-being, Tridimensional model, Life satisfaction, Harmony in life, Positive affect, Negative affect, Multidimensional Item Response Theory

## Abstract

**Background:**

Happiness is often conceptualized as subjective well-being, which comprises people's evaluations of emotional experiences (i.e., the affective dimension: positive and negative feelings and emotions) and judgements of a self-imposed ideal (i.e., the cognitive dimension: life satisfaction). Recent research has established these two dimensions as primary parts of a higher order factor. However, theoretical, conceptual, and empirical work suggest that people's evaluations of harmony in their life (i.e., the sense of balance and capacity to behave and adapt with both acceptance and flexibility to inter- and intrapersonal circumstances) constitutes a third dimension (i.e., the behavioral dimension). This tridemensional conceptualization of subjective well-being has recently been verified using Unidimensional Item Response Theory (UIRT) and Classical Test Theory (CTT). Here, we use a recently developed and more robust approach that combines these two methods (i.e., Multidimensional Item Response Theory, MIRT) to *simultaneously* address the complex interactions and multidimensionality behind how people feel, think, and behave in relation to happiness in their life.

**Method:**

A total of 435 participants (197 males and 238 females) with an age mean of 44.84 (*sd* = 13.36) responded to the Positive Affect Negative Affect Schedule (10 positive affect items, 10 negative affect items), the Satisfaction with Life Scale (five items), and the Harmony in life Scale (five items). We used Bifactor-Graded Response MIRT for the main analyses.

**Result:**

At the general level, each of the 30 items had a strong capacity to discriminate between respondents across all three dimensions of subjective well-being. The investigation of different parameters (e.g., marginal slopes, ECV, IECV) strongly reflected the multidimensionality of subjective well-being at the item, the scale, and the model level. Indeed, subjective well-being could explain 64 % of the common variance in the whole model. Moreover, most of the items measuring positive affect (8/10) and life satisfaction (4/5) and all the items measuring harmony in life (5/5) accounted for a larger amount of variance of subjective well-being compared to that of their respective individual dimensions. The negative affect items, however, measured its own individual concept to a lager extent rather than subjective well-being. Thus, suggesting that the experience of negative affect is a more independent dimension within the whole subjective well-being model. We also found that specific items (e.g., “Alert”, “Distressed”, “Irritable”, “I am satisfied with my life”) were the recurrent exceptions in our results. Last but not the least, experiencing high levels in one dimension seems to compensate for low levels in the others and vice versa.

**Conclusion:**

As expected, the three subjective well-being dimensions do not work separately. Interestingly, the order and magnitude of the effect by each dimension on subjective well-being mirror how people define happiness in their life: first as harmony, second as satisfaction, third as positive emotions, and fourth, albeit to a much lesser degree, as negative emotions. Ergo, we argue that subjective well-being functions as a complex biopsychosocial adaptive system mirroring our attitude towards life in these three dimensions (A: affective dimension; B: behavioral dimension; C: cognitive dimension). Ergo, researchers and practitioners need to take in to account all three to fully understand, measure, and promote people's experience of the happy life. Moreover, our results also suggest that negative affect, especially regarding high activation unpleasant emotions, need considerable changes and further analyses if it is going to be included as a construct within the affective dimension of a general subjective well-being factor.

## 1 Introduction

For almost over 40 years, happiness has been conceptualized as subjective well-being [[1], [2], [3], [4]]. Subjective well-being consists of a person's evaluations about their own experience of emotional reactions that stem from the nervous system (i.e., the affective dimension: positive affect and negative affect) and judgements of their life in relation to a psychological self-imposed ideal (i.e., the cognitive dimension: life satisfaction). In other words, subjective well-being is comprised of two distinct dimensions that indicate how we feel and think about our life. It has been argued that individual reactions to objective life circumstances at different levels are reflected by these subjective evaluations in ways that alternative approaches cannot measure—for example, objective-list-based models of well-being that specify “all” the critical ingredients that are required for a person to have a good life [5]. Indeed, its subjective nature captures individual differences in how people weigh objective circumstances depending on how their nervous system reacts and their own goals, values, and culture [6,7]. Thus, subjective well-being is probably one of the best proxies for measuring, studying, and understanding human well-being and the impact of events, interventions, or public-policy decisions on quality of life [6]. Nevertheless, there is still a debate about major methodological and theoretical questions that need to be addressed [6,[8], [9], [10], [11], [12], [13], [14], [15]].

### 1.1 Methodological issues: unidimensionality or multi-dimensionality

One of the major current issues is that researchers seem to use different approaches, usually unstated or even discussed, for the operationalization of subjective well-being and its dimensions [16]. As detailed by Busseri and colleagues [16], some of the main approaches are (a) measuring just one dimension (i.e., either the affective or the cognitive dimension): as the indicator of subjective well-being; (b) examining both dimensions separately, which is usually motivated through scientific evidence suggesting that the two subjective well-being dimensions correlate differently with the same variables (e.g., personality, life events, and demographic characteristics); (c) using a composite subjective well-being score by standardizing positive affect, negative affect, and life satisfaction and then subtracting the negative affect score from the sum of positive affect and life satisfaction scores, thus, assuming that all constructs are equally important; and finally yet most importantly (d) accounting for the presence of a general subjective well-being factor (i.e., higher order latent factor) to which the variance in each subjective well-being construct is attributable to. In most current research, however, it is unclear whether the results of the first three of these approaches relate to the common variance in subjective well-being or to the unique variance in each specific construct (i.e., positive affect, negative affect, and life satisfaction). The fourth approach, on the other hand, accounts for both a common source variance and a unique source variance in each construct.

In six longitudinal studies, Busseri and colleagues [16] demonstrated (I) that the majority of explained variance in life satisfaction belongs to the common variance in subjective well-being, rather than to the specific variance of life satisfaction; (II) that positive affect explained about equal amount of variance regarding both the common variance in subjective well-being and its own specific variance; and (III) that negative affect explained more of its own specific variance compared to the common variance in subjective well-being, see also [[17], [18], [19], [20], [21]]. Hence, this new line of research does not only confirms Diener's [2] theorized dual dimensionality of subjective well-being and its three distinctive constructs (i.e., positive affect, negative affect, and life satisfaction), but suggests a new methodological principle in which the subjective well-being dimensions are primary parts of a whole—that is, subjective well-being is multi-dimensional rather than an unidimensional single construct (cf. [22] who shows similar issues for depression).

### 1.2 Theoretical issues: a third subjective well-being dimension

Another major issue concerns how to conceptualize and measure the two subjective well-being dimensions. Regarding affect, for example, we know that one of the major predictors of subjective well-being, Neuroticism, has a larger effect on subjective well-being when the affect dimension is measured as how often emotions are experienced in contrast to how intensive [[23], [24], [25]]. Moreover, people who are regarded as happy, experience intensive positive emotions only about 2.6 % of the time, but they feel contented or mildly happy very frequently [[26], [27], [28]]. Hence, the consensus is that how (in)frequently a person experiences positive and negative affect, rather than how intensively, are better conceptualizations of the two constructs of the affective dimension [23,[29], [30], [31]]. Regarding life satisfaction, however, researchers have recently questioned if it is the only way to conceptualize the cognitive dimension. In short, the argument is that the focus on life satisfaction in subjective well-being research is related to both Western and contemporary ways of looking at happiness, which are not necessarily attune to how Eastern and antique civilizations and people in general conceptualize happiness and the good life [15,[32], [33], [34], [35], [36]].

Indeed, ancient civilizations have emphasized the need of harmony in life as one part of the happy life. As explained elsewhere, for the Greek, harmony referred to a person's ability to maintain balance and serenity in both happy and challenging times [34]. Moreover, modern definitions of happiness as a psychological concept describe it as the sense of a harmonious and positive connection within the self and between the self and others [37]. In other words, in contrast to life satisfaction, which focuses on the self's ideals and wishes, the sense of harmony in life implies homeostasis or the regaining of balance through the person's capacity to behave and adapt with both acceptance and flexibility to circumstances in the different planes of life, both within and outside the self [15]. Ergo, harmony might be a third subjective well-being dimension addressing people's social behavior [15,38,39].

The concept of harmony is distinctive from life satisfaction, not only through theoretical and philosophical definitions, but also by how people define and differentiate them from each other. Quantitative semantics^1^ studies, for example, show that people use words such as, peace, balance, unity, agreement, calm, mediation, cooperation, tolerant, nature, and forgiveness, when they describe how they pursue harmony in life; while they use words such as, job, money, achievement, education, success, wealth, house, and gratification when they describe how they pursue life satisfaction. Consequentially, life satisfaction is related to people's levels of self-centeredness, self-interest, independence, mastery, and personal achievement; while harmony in life is associated to selflessness, psychological balance, flexibility as well as the sense of relatedness, interconnectedness, and peace [35,40]. Likewise, qualitative and quantitative studies across individualistic and collectivistic cultures (e.g., Australia, Croatia, Germany, Italy, Portugal, Spain, and South Africa) show that lay people primarily define happiness as psychological balance and harmony—life satisfaction and emotions are only used as subordinate definitions [34]. In addition, although harmony in life is strongly correlated to life satisfaction (*r* = 0.76), factor analytic studies of the scales that are commonly used to measure them, show that they are two distinctive constructs [32,33,35]. Related to this, a study conducted during the COVID-19 pandemic showed that inner harmony was a better predictor of less negative health problems, such as, stress, anxiety, and general distress, than other well-being factors [41]. Hence, harmony in life is semantically and psychometrically different than the concept of life satisfaction and might not only be the third dimension of happiness that has been forsaken in most research, but also the most essential one.

### 1.3 A tridimensional model of subjective well-being

**Fig. 1:**
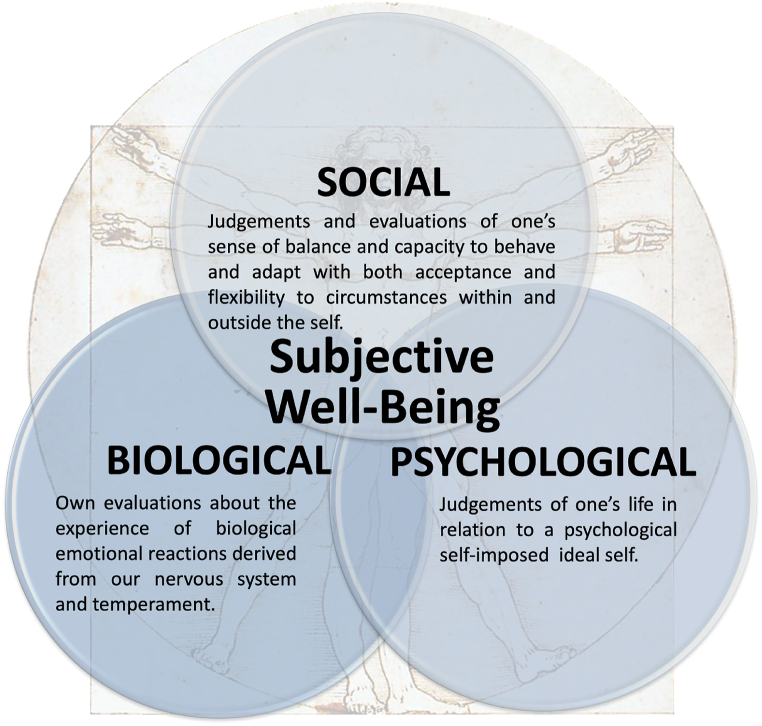
Subjective Well-Being as a tridimensional construct consisting of positive affect and negative affect (Biological: affective dimension), life satisfaction (Psychological: cognitive dimension), and harmony in life (Social: behavioral dimension).

To this date, most research has neglected that subjective well-being is probably better measured as a higher order factor and that harmony in life is a construct that represents its behavioural or social dimension. Certainly, subjective well-being should be considered as the holistic apprehension or attitude towards happiness in our life in three dimensions (see Fig. 1): evaluations of biological emotional reactions (i.e., the affective dimension), judgements of a psychological self-imposed ideal (i.e., the cognitive dimension), and the apprehension of one's sense of balance and capacity to behave and adapt with both acceptance and flexibility to intra- and interpersonal relations and circumstances (i.e., the behavioral dimension). In this line of thinking, we have tested this tridimensional model of subjective well-being by investigating each construct at the item, at the response, and at the scale level using unidimensional Item Response Theory (UIRT) techniques and by investigating its structure at the model level using both mainstream and more advanced Classical Test Theory (CTT) techniques. The analyses were conducted using the most common measures for each construct in two different populations (i.e., the Positive Affect Negative Affect Schedule, the Satisfaction with Life Scale, and the Harmony in Life Scale).

Our results indicated large residuals that were significantly correlated, thus, suggesting the existence of a latent multidimensional structure in the data rather than a clear unidimensional nature. Moreover, the further UIRT analyses showed that (a) the affective dimension, especially low levels of negative affect, were less accurately measured, while both the cognitive and behavioral dimension were covered to an equal degree by the measures we tested; (b) that the subjective well-being measures had less reliability for individuals with extreme scores in the four constructs; and (c) that specific items (e.g., the item “Alert” measuring positive affect; the item “I fit in well with my surroundings” measuring harmony in life) might need to be modified or replaced. Nevertheless, despite the robustness of UIRT regarding the violations against the assumptions of unidimensionality and local independence, the complex interactions between the four constructs within the three subjective well-being dimensions required statistical methods that could handle the multidimensional nature found in the data [39]. Therefore, using a second sample [38], we applied CTT to scrutinize the multidimensionality patterns of subjective well-being by testing several models: (a) a standard unidimensional model in which all items measuring each of the four constructs (i.e., positive affect, negative affect, life satisfaction, and harmony in life) loaded only on subjective well-being as one common latent factor; (b) four separate orthogonal constructs in an unidimensional model; (c) a multidimensional model with intercorrelated constructs without subjective well-being as a common latent factor; (d) a higher order multidimensional factor model where the four constructs as lower order factors were correlated with subjective well-being as the higher order factor (i.e., subjective well-being accounting for the commonality shared by the lower order factors); and last (e) a bifactor model in which subjective well-being was a single general factor and each construct were orthogonal secondary dimensions that had an effect on each of its respective items (i.e., nested multidimensional factors or a hierarchical factor model).

The first two models were unable to measure the multifaced nature of the data, which was indicated by low fit indices and large residuals. Regarding the multidimensional models, the third model (i.e., the multidimensional correlated model) indicated a good model fit, but it included only the four correlated constructs without any information about the overall single latent subjective well-being factor. Additionally, despite the fourth model's (i.e., higher order multidimensional factor model) acceptable fit indexes, it still had some limitations. It did, for example, not provide a full understanding of the proposed tridimensional structure, because the effect of the general latent subjective well-being factor on each item was mediated by the four specific constructs. In other words, there was no direct relationship between subjective well-being (i.e., the first order factor) and each of the items measuring the different subjective well-being constructs. The fifth model (i.e., bifactor model), on the other hand, showed the best fit indices compared to all other four models. Importantly, even though all three multidimensional models (third, fourth and fifth model) seemed to fit the data better than the unidimensional models (first and second model), only the bifactor model (fifth model) captured and reflected the multidimensional nature of subjective well-being.

In essence, in the bifactor model (I) about 64 % of the variance of individuals' subjective well-being was explained by this general factor, (II) the items measuring the affective dimension reflected a mixture of participants' levels of subjective well-being and levels of each respective type of affective construct (i.e., positive affect and negative affect), but contributed significantly more (positive affect: 48 %; negative affect: 49 %) to their respective specific affective construct than to subjective well-being (positive affect: 25 %; negative affect: 32 %); (III) the items measuring the cognitive dimension contributed to a larger degree to participants’ level of subjective well-being (72 %) than to life satisfaction itself (22 %); (IV) the items measuring the behavioral dimension contributed to an even larger degree to subjective well-being (98 %) than to harmony in life itself (0 %); and (V) most of the same items as in the UIRT study needed to be modified or replaced [38,39].

### 1.4 The present study: Multidimensional Item Response Theory (MIRT)

Until this point, in two separate studies, we have validated our proposed tridimensional model of subjective well-being at the item level, at the item response level, at the scale level, and at the model level. We have not only replicated past seminal work that laid the ground for subjective well-being as a higher order construct in which each lower order construct contributes to different degrees, but also added and verified harmony in life as the behavioral dimension that is at the core of people's happiness. We therefore argue that subjective well-being is a complex adaptive system in which the different dimensions contribute to different degrees and compensate for one another.

Despite the robustness of these prior research, ours and that of others, both CTT and UIRT approaches still have many limitations. One of these being that CTT statistics estimates are assumed to be equal for all individuals, which makes them heavily dependent on the sample's particular characteristics—e.g., size and sociodemographic characteristics [42,43]. We compensated this short coming by using UIRT techniques in a separate study—in UIRT, the location estimates of each person's response are invariant with respect to the specific measure of the construct, thus, the precision of UIRT estimates is at the individual level and not just at the group level as in CTT [42,44,45]. Moreover, UIRT also overcomes the limited information from correlation matrices found in factor analysis because it explains an individual's observed response pattern by tracing the interaction of their level in a specific subjective well-being construct with various item-level characteristics, such as, “discrimination” or the probability of randomly choosing the answer that is accurate for the participant [46,47].

Nevertheless, the proposed tridimensional subjective well-being model implies complex interactions of items measuring each construct, complex interactions between each construct, and complex interactions to subjective well-being as general factor. What is even more, all these complex interactions are happening simultaneously in people's train of thought when they evaluate their emotions, life satisfaction, and harmony in life. Thus, further validation might require techniques that take advantage of a combination of UIRT and CTT, such as Multidimensional Item Response Theory (MIRT) [[48], [49], [50]]. MIRT models, an extension of UIRT, have the advantages of UIRT models and also describe the interaction of vectors of constructs with the characteristics of test items rather than assuming a single construct parameter [48,50]. MIRT enables to model and develop an intuitive understanding regarding the vectors of more than one underlying dimension, which helps to explain the interaction of items' properties (e.g., discriminating between levels of several different constructs) and the respondent's capacity and characteristics regarding these multiple constructs—the model can be classified in relation to the interaction between and within the subjective well-being general factor and the constructs compensatory dynamic that is accounted for using MIRT allow high proficiency on one construct to compensate for low proficiency on the other constructs and vice versa [42,50]. This is extremely important for the present study, because the capacities and characteristics that influence responses to a multidimensional concept can vary both between and within the individual—for example, two individuals might have high levels of life satisfaction but differ in their levels of harmony in life, which also means that while one of this individuals is high in both life satisfaction and harmony in life, the other has high levels of life satisfaction but low levels of harmony in life.

Here we use MIRT to investigate the tridimensionality of the cognitive processes behind people's responses to each of subjective well-being's three dimensions and its four constructs. In contrast to CTT, which considers only covariances between items, and UIRT, which considers only covariances between items and individuals for one construct at a time; MIRT allow us to account for both multidimensional item difficulty and multidimensional item discrimination across all subjective well-being dimensions. Moreover, by applying MIRT we can test the structures of the dimensions by treating them as different latent dimensions in order to assess the multidimensionality of the model in which the items reflect the different evaluation processes with regard to positive affect, negative affect, life satisfaction, and harmony in life. Lastly but most importantly, throughout MIRT we can determine the strength of the relationships between the four constructs within the three subjective well-being dimensions. Using this more robust technique, we expected to replicate, *simultaneously*, our past findings about the tridimensionality of subjective well-being when we used separate techniques (i.e., CTT and UIRT) in two different studies and samples.

## 2 Method

### 2.1 Ethics statement

Ethics approval was not required as per national regulations. The study was performed in accordance with the ethical standards of the 1964 Helsinki declaration and its later amendments. All participants were provided with the relevant information about the research, such as, anonymity, voluntary participation, and that they had the choice to terminate the survey at any time without any repercussions. Their consent was obtained throughout survey completion.

### 2.2 Participants and data collection procedure

All participants were recruited throughout Amazon's Mechanical Turk,^2^ originated from the USA, and had English as their native language. Those who accepted to participate received $1.00 as compensation. We included three questions (e.g., This is a control question, please answer “neither agree nor disagree”) to control for the participants attention to the items in the survey and controlled for automatic responses by electronically monitoring the time it took for participants to take the whole test. We removed all data from participants who responded erroneously to one or all the control questions and from those who responded under 10 min (about 57 %). Hence, the final sample consisted of 435 participants (197 males and 238 females) with an age mean of 44.84 (*sd* = 13.36); in which 10.30 % reported being divorced, 2.80 % widowed 12.40 % living with another, 49.40 % married, 0.90 % separated, and 24.10 % single. Moreover, about 71.56 % were employed for wages, 16.06 % were self-employed, 3.44 % were out of work, 3.90 % were homemakers, 0.46 % were students, 3.90 % were retired, and 0.69 % reported being unable to work.

### 2.3 Measures

#### 2.3.1 Affective dimension

The Positive Affect Negative Affect Schedule, PANAS [51,52], asks participants to rate to what extent they have experienced 10 positive (e.g., “Alert”) and 10 negative (e.g., “Irritable”) feelings and moods during the last week, using a 5-point Likert scale (1 = *very slightly* or not at all, 5 = *extremely*). Importantly, for the analyses the negative affect scores were reversed since the these items are negatively related to subjective well-being.

#### 2.3.2 Cognitive dimension

The Satisfaction with Life Scale, SWLS [3,4], comprises five items (e.g., “I am satisfied with my life”) with a 7-point Likert scale (1 = *strongly disagree*, 7 = *strongly agree*).

#### 2.3.3 Behavioral dimension

The Harmony in Life Scale, HILS [35] comprises five statements (e.g., “I am in harmony”) with a seven-point Likert scale (1 = *strongly disagree*, 7 = *strongly agree*).

### 2.4 Statistical treatment

In the present study, the basic assumptions for using IRT models (i.e., appropriate dimensionality, local independence, and monotonicity) were all satisfactorily met—see Supplementary Material for a general description of these parameters and a detailed description of the testing of these assumptions in the present study. After establishing that our data complied with these assumptions, we proceeded to choose an appropriate MIRT technique. Based on the studies presented in the Introduction, we decided to use Bifactor-Graded Response MIRT (Bifactor-GR MIRT)—a especial type of Bifactor Polytomous MIRT that allowed us to test the proposed tridimensional model by treating each subjective well-being construct (i.e., positive affect, negative affect, life satisfaction, and harmony in life) as orthogonal subdimensions, that is, uncorrelated with each other. Moreover, this technique allowed us to freely estimate the association between the general subjective well-being factor and each of the 30 items in the model without being dependent on any mediating sub-paths throughout any of the four constructs. We used R version 3.6.3, SPSS version 26, and Microsoft Excel to analyse the data.

## 3 Results

**Fig. 2:**
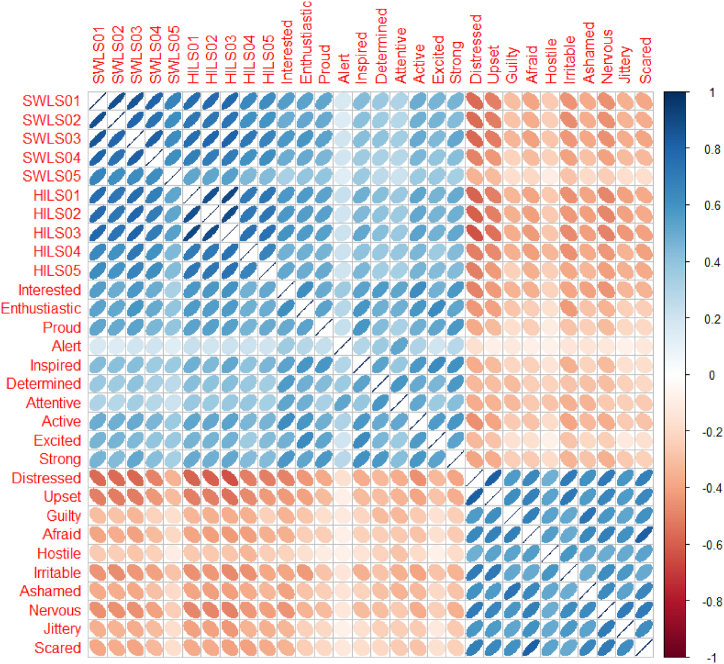
Matrix scatter plots of correlations between the 30 items in the proposed tridimensional model of subjective well-being (*n* = 435). Note: The color of the ellipses indicates negative (towards the red spectrum) and positive correlations (towards the blue spectrum) between items—the stronger the color, the stronger the correlation. The size of the ellipses also indicates the strength of the correlation, the more elongated the ellipse is (i.e., the more closed the ellipse), the closest to 1 the correlation is. SWLS = Satisfaction with Life Scale; HILS = Harmony in Life Scale. SWLS01: “In most ways my life is close to my ideal”; SWLS02: “The conditions of my life are excellent”; SWLS03: “I am satisfied with my life”; SWLS04: “So far, I have gotten the important things I want in life”; and SWLS05: “If I could live my life over, I would change almost nothing”; HILS01: “My lifestyle allows me to be in harmony”; HILS02: “Most aspects of my life are in balance”; HILS03: “I am in harmony”; HILS04: “I accept the various conditions of my life”; and HILS05: “I fit in well with my surroundings”. (For interpretation of the references to color in this figure legend, the reader is referred to the Web version of this article.)

As part of the explorative analysis, Fig. 2 displays the negative (in red spectrum) and positive (in blue spectrum) correlations between all 30 items in our model—the stronger the color, the stronger the correlation. All the correlations were significant at *p* < .01 and most correlations ranged from −0.63 to 0.90. The strongest correlation (*r* = 0.90), both within and between subscales, was the correlation between the harmony in life item “My lifestyle allows me to be in harmony” (HILS01) and the life satisfaction item “I am satisfied with my life” SWLS03 and the weakest (*r* = 0.14) was between the positive affect item “Alert” and the life satisfaction item “If I could live my life over, I would change almost nothing” (SWLS05). In other words, most items within each of the four specific subscales were highly intercorrelated and correlated across the other subscales. Thus, reflecting general factor and subfactor saturation [53]. As in past studies using CTT approaches, the only exception was the positive affect item “Alert”, which was only weakly correlated to items within its specific subscale and also with items within the other subscales.

Table 1 summarizes all the parameter estimates calculated using Bifactor-GR MIRT (i.e., slope, intercept, multidimensional item discrimination, multidimensional item location, marginal factor loading for general trait, marginal factor loading for specific trait, marginal slope for general trait, marginal slope for specific trait, communality, proportion of common variance across all items explained by general trait, proportion of common variance across all items explained by specific trait, item explained common variance for the general trait, and item explained common variance for specific trait). The item discrimination parameters (i.e., conditional and marginal discrimination parameters) of the multidimensional latent factors in the model had an equivalent interpretation regarding discrimination power of the item as they are in the unidimensional latent trait model [42,50]. Nevertheless, as recommended [42], we also calculated the multidimensional item discrimination parameter, since it reflects the vectors of the power of discrimination for more than one latent factor in the multidimensional latent space. In other words, the multidimensional item discrimination parameter, regardless of the number of latent factors, is conceptually analogous to the unidimensional discrimination item and can be interpreted similarly, but the multidimensional item discrimination parameter is conditioned by the discrimination parameter for each of the different latent dimensions [42,50]. Mathematically, it is the square root of the sum of squared discrimination parameters (conditional slopes) for each of these underlying dimensions. Importantly, as for unidimensional models, the greater the item's discrimination capability, the greater the item's multidimensional information is in a particular direction [42,54]. These analyses showed that 29 items out of the 30 in the model, with the exception of the positive affect item “Alert” which had a relatively low value (1.13), had very high values with regard to multidimensional item discrimination parameters (*mean* = 3.48). Thus, indicating that each item had a strong vector with the capacity to differentiate and discriminate between different levels of the latent factor for respondents across all dimensions in the latent factor space [42,50,54].

**Table 1:**
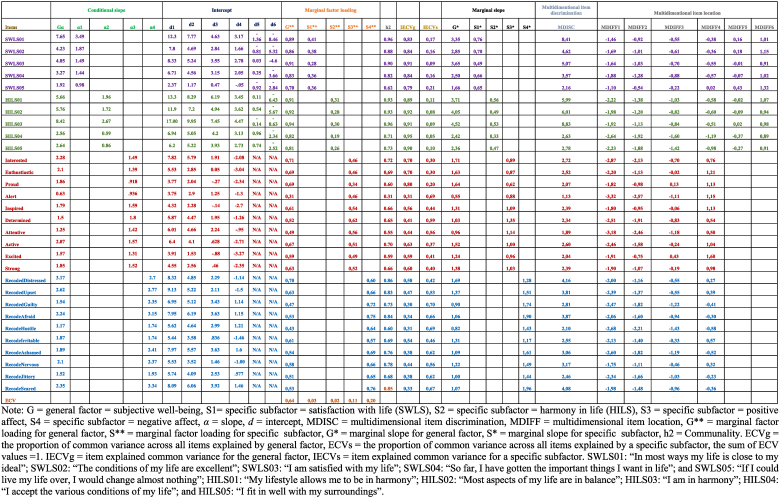
Items parameter estimates for the proposed tridimensional model of subjective well-being using Bifactor-GR MIRT (*n* = 435).

Furthermore, the result showed that the mean of the conditional slope in the general latent subjective well-being factor was 2.81, 1.39 for positive affect, 2.45 for negative affect, 1.85 for life satisfaction, and 1.56 for harmony in life. Overall, participants' subjective well-being (i.e., the general factor) had a strong effect on participants’ responses to each of the items within the model; especially across the life satisfaction and harmony in life items. Again, the only exception was the positive affect item “Alert”, which had a low conditional slopes value (0.63). Moreover, the items measuring life satisfaction and harmony in life contributed to a higher degree to the general factor, rather than to their own subfactors. The harmony in life item: “I accept the various conditions of my life” (HILS04) had the lowest conditional slope (0.59) in the specific latent harmony in life subfactor. Most of the positive affect items contributed more to the general subjective well-being factor compared with their contribution to their own positive affect subfactor, with the exception of the positive affect items “Alert”, “Determined”, and “Attentive”, which contributed more to their own positive affect subfactor than to the general subjective well-being factor. Most of the negative affect items contributed more to their own negative affect subfactor than to the general subjective well-being factor, with the exception of the negative affect items “Distressed” and “Irritable”, which contributed more to the general subjective well-being factor than to their own negative affect subfactor. See Table 1 for the details.

Nevertheless, since the probability of item response pattern depends on the conditional interaction among the general factor and the other subfactors, we used marginal slopes to simplify and clarify this conditional interaction [55]. To calculate marginal slopes for both the general subjective well-being factor and its subfactors, we first calculated the marginal factor loadings for both the general factor and subfactors, and then we calculated the unexplained item variances for both the general factor and subfactors [55]. The mean of the marginal slope for the general latent factor (i.e., subjective well-being) was 1.85 and for the specific latent subfactors were 1.55 for negative affect, 0.98 for positive affect, 0.65 for life satisfaction, and 0.47 for harmony in life.^3^ Overall, there was shrinkage in marginal slopes compared with conditional slopes. In other words, the inflation of the conditional slopes was a consequence of the conditional interactions between the general subjective well-being factor and the four subfactors. However, in general, the results still indicated that the general subjective well-being factor had a strong effect on the item responses across all items of the model especially across items measuring life satisfaction and harmony in life—with the exception of the positive affect item “Alert”, which had a low value (0.55). The items measuring life satisfaction and harmony in life contributed to a higher degree to the general subjective well-being factor compared with their contribution to their own subfactor. Additionally, the Satisfaction with Life Scale item “I am satisfied with my life” (SWLS03) had low discrimination (0.49) with respect to its own life satisfaction subfactor. Furthermore, most of the items in the Harmony in Life Scale had low discrimination (ranging from 0.33 to 0.56) to its own harmony in life subfactor. Regarding the Positive Affect Negative Affect Schedule, most of the positive affect items, except the items “Alert”, “Determined” and “Attentive”, contributed more to the general subjective well-being factor than to the positive affect subfactor; while most of the negative affect items, except the items “Distressed” and ”Irritable”, contributed more to the negative affect subfactor than to the general subjective well-being factor. See Table 1 for details.

In the present study, the bifactor model yielded an explained common variance^4^ for the general subjective well-being factor (ECVg) equal to 64 % of the common variance in the observed scores in all 30 items within the proposed tridimensional model. Ergo, 36 % of the common variance in the observed scores in all 30 items of the proposed tridimensional model was spread across the four subfactors (ECVs for positive affect was 11 %, ECVs for negative affect was 20 %, ECVs for life satisfaction was 3 %, and ECVs for harmony in life was 2 %). In general, this high value of ECVg suggests that the proposed model has a strong general latent dimension. See Table 1 for details.

Next, we inspected the explained common variance at the item level^5^ for the general factor (IECVg) and the subfactors (IECVs). Regarding positive affect, IECVg ranged from 41 % to 80 % and IECVs ranged from 20 % to 69 %. More specifically, seven out of the 10 positive affect items contributed more to the loadings between them and the general subjective well-being factor rather than to the loadings between them and the positive affect subfactor— the positive affect items that contributed more to its own subfactor were the items “Alert”, “Determined”, and “Attentive”. Concerning the negative affect, IECVg ranged from 33 % to 58 % and IECVs ranged from 42 % to 70 %. Here, eight out of the 10 negative affect items contributed more to the negative affect subfactor than to the general subjective well-being factor — the negative affect items that contributed more to the general factor were the items “Irritable” and “Distressed”. The inspection of the items measuring life satisfaction resulted in IECVg values ranging from 79 % to 91 % and IECVs values ranging from 9 % to 21 %, while the inspection of the items measuring harmony in life resulted in IECVg values ranging from 89 % to 95 % and IECVs values ranging from 5 % to 11 %. Thus, indicating that the loadings between the items measuring life satisfaction and the general subjective well-being factor and between the items measuring harmony in life and the general subjective well-being factor explained a clear majority of the percentage of the expected common variance (between 79 % and 95 %), while much less (between 5 % and 21 %) was explained by the loadings between the items measuring life satisfaction and its own subfactor and between the items measuring harmony in life and its own subfactor. See Table 1 for details.

Regarding intercept and multidimensional item difficulty, it is first important to mention that, since the item intercept term includes both multidimensional item difficulty and multidimensional item discrimination parameters (i.e., the interaction between the multidimensional item difficulty and multidimensional item discrimination; Intercept = - (MDIFF*MDISC), it cannot be interpreted as a multidimensional item difficulty parameter. In other words, we cannot offer an easy interpretation of multidimensional item difficulty using the intercept item parameter, thus, we also calculated the multidimensional item difficulty parameter as a single value that represents the difficulty of the item being tested. In general, both a negative and/or a low value of the multidimensional item difficulty parameter indicates that this item requires low levels of the underlying trait to have a high probability (greater than 0.5) of being chosen and to correspond correctly to the respondent's actual level in that specific trait. In other words, multidimensional item difficulty parameter in multidimensional space of traits is interrupted just as the parameter of difficulty on the continuum in UIRT model. In short, we found that the multidimensional item difficulty parameters were between −3.32 on response 1 and 1.60 on response 5 for the positive affect scale, −2.68 on response 1 and 0.57 on response 5 for the negative affect scale, −3.88 on response 1 and 1.32 on response 7 for the Satisfaction with Life Scale, and −2.64 on response 1 and 1.07 on response 7 for the Harmony in Life Scale, (See Table 1 for details). It should be considered that the negative affect items were recoded when we applied the Bifactor-GR MIRT. Hence, negative affect had the highest estimated multidimensional item difficulty on response 5 (2.68) and the lowest on response 1 (−0.57).

Before going further with the results is important to mention that GR UIRT models generate an item category curve, while Bifactor-GR MIRT models yield item responses category surfaces which reflect the probability of each category responses that is conditional on multiple latent traits, not single traits as in GR UIRT. In other words, in the unidimensional models there is a single point where the probability of choosing responses is equal to 0.50, while in a multidimensional model there is an *inflexion line* for which the probability of choosing a response is equal to 0.50 in a multidimensional space (see Fig. 2ab for an example of this important difference between GR MIRT, 2a, and GR UIRT, 2b). In general, the results in the present study using Bifactor-GR MIRT regarding item category response surface revealed that when both the general subjective well-being latent factor (*θ*1) and the specific four subfactors (*θ*2) increase, the probability of choosing the lowest category response (1) decreases and the probability of choosing the highest response category increases (5 for positive and negative affect and 7 for both life satisfaction and harmony in life). The probability of choosing any of the intermediate category responses (2, 3 and 4 for positive and negative affect and 2, 3, 4, 5 and 6 for life satisfaction and harmony in life), on the other hand, increases when the probability of choosing responses to the first alternative decrease, only to decrease again when the probability to choose the highest response category increases (i.e., the general subjective well-being latent factor, *θ*1; the specific four subfactors, *θ*2). Thus, this result reflected that the probability of the first response (1) decreased monotonically when there is an increase in any one or any combination of the respondents' latent trait, while the probability of the last response (5 for positive and negative affect, 7 for life satisfaction and harmony in life) increased monotonically when there is an increase in any one or any combination of the respondents’ latent trait in our proposed tridimensional model of subjective well-being.

**Fig. 3:**
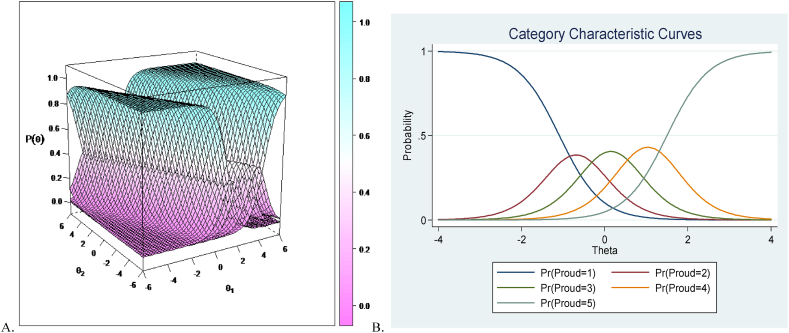
Example of item category response surface (positive affect item “Proud”) using Bifactor-GR MIRT (A), and category characteristic curves for the same item using UIRT (B; from Nima et al., 2020b). Note: *p* = probabilities of each response; *θ* = theta, which represents a person's latent subfactor that has been standardized to follow standard normal distribution; *θ1* = theta for the general factor (subjective well-being); *θ2* = theta for the subfactor (i.e., positive affect). The graph regarding item category response curves reflects the probabilities of each category responses (i.e., the probabilities of each of the five category responses,1, 2, 3, 4 and 5) conditional on the level of only one latent subfactor. The graph (B) shows the location where the next category becomes more likely (not 50 %), that is, the points on the curve where the adjacent categories cross represent transitions from one response point to the next.

**Fig. 4:**
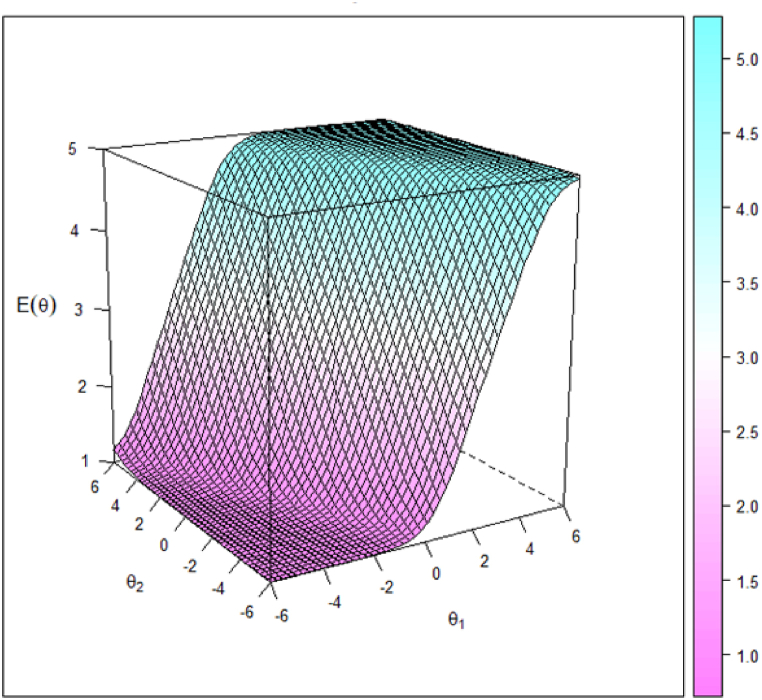
Expected item score surface for an example item (positive affect item “Proud”) using Bifactor-GR MIRT (*n* = 435). Note: *θ1* = theta for the general factor (subjective well-being); *θ2* = theta for the subfactor (i.e., positive affect).

As the next step in our analyses, we explored the expected item response score, which is a descriptive function that is unique to polytomous models used to compute the expected test item score for a respondent with given latent traits. It is a useful tool to examine the expected score function in addition to the probability of selecting a certain response. We found a similar pattern for all 30 items in the model. Due to space issues, we only show the results for the positive affect item “Proud”, which expected score represents the five possible response categories for this item (i.e., ranging 1 to 5). In our data, the expected score was near to 5 when levels on both the subjective well-being factor and the positive affect subfactor were high. Conversely, the expected score decreases near to 1 when levels on both the subjective well-being factor and the positive affect subfactor were low. Nevertheless, high levels in subjective well-being in conjunction with low levels in positive affect could yield a response of 5 in the item “Proud”, while low levels in subjective well-being in conjunction with high level in positive affect could also yield a response of 5 in the item “Proud” (see Fig. 3). This result probably reflects the compensatory multidimensional nature of the proposed tridimensional model of subjective well-being; where a person's high level in one latent factor or subfactor might compensate for a low level in the other factor/subfactor, and vice versa. Fig. 4 depicts the expected item response score for an example item (positive affect item “Proud”).

**Fig. 5:**
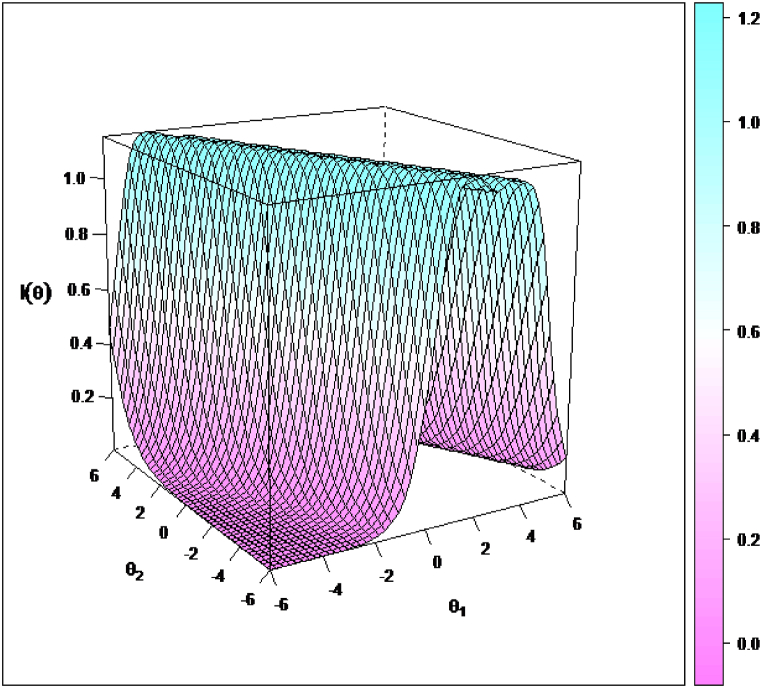
Multidimensional Item Information Surface for the positive affect item “Proud” using Bifactor-GR MIRT (*n* = 435).

**Fig. 6:**
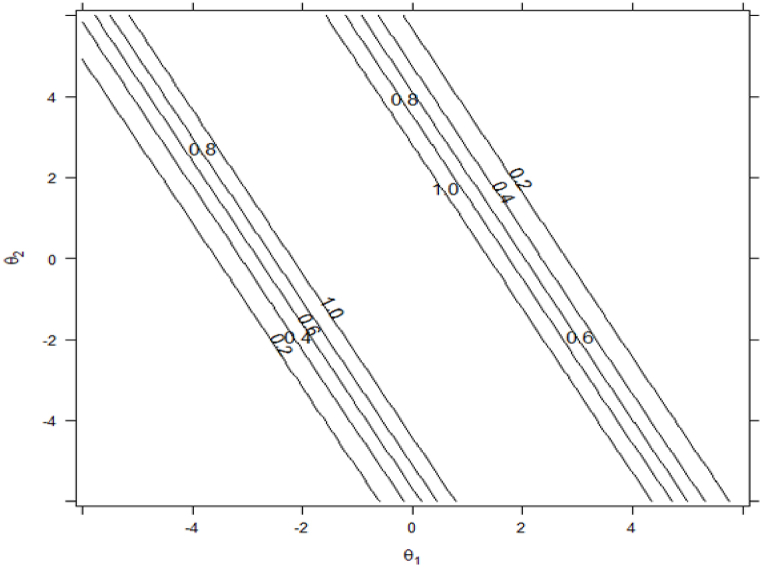
Contour plot of Multidimensional Item Information Surface for the positive affect item “Proud” using Bifactor-GR MIRT (*n* = 435).

**Fig. 7:**
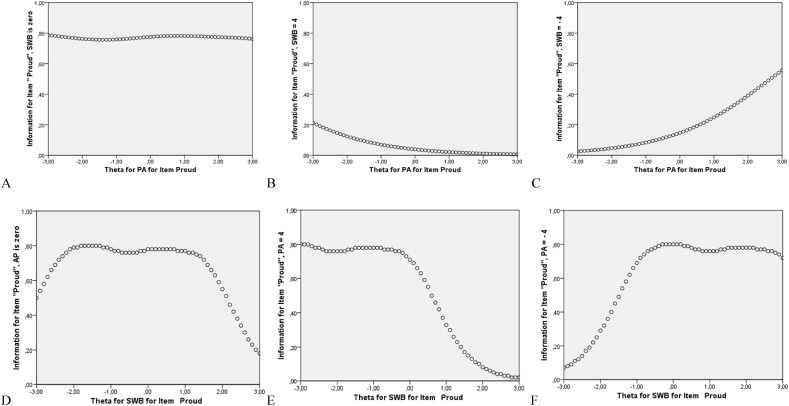
Multidimensional Item Information Function (based on marginal slopes) for the positive affect item “Proud” using in Bifactor-GR (*n* = 435). Note: (A) the amount of information on positive affect (PA) at the value (*θ* = 0.00) for subjective well-being (SWB); (B) the amount of information on PA at the value (*θ* = 4.00) for SWB; (C) the amount of information on PA at the value (*θ* = −4.00) for SWB; (D) the amount of information on SWB at the value (*θ* = 0.00) for PA; (E) the information on SWB at the value (*θ* = 4.00) for PA; (F) the amount of information on SWB at the value (*θ* = −4.00) for PA.

In general, with some modifications, the results regarding multidimensional item information^6^ surface showed that the 30 items in our proposed model could provide a lot of information to cover the ideal range along the general subjective well-being latent factor and the four latent subfactors. Due to lack of space, we only show here one example using the positive affect item “Proud”. This item provided the most precise measure of respondent's latent factor and subfactor (*θ*1 for subjective well-being, *θ*2 for positive affect) along the shape of the high ridge from about −3.00 to 2.50 for subjective well-being and 6.00 to −6.00 for positive affect in the multidimensional latent traits space (see Fig. 4). To simplify the result of the multidimensional item information surface, we used a contour plot (i.e., a graphical technique for representing a tridimensional surface). The inspection of the contour plot showed that the item “Proud” was relatively ineffective to provided information for respondents in the lower and upper triangles that are bounded by information = 0.20 contour lines. The wide white area in the middle of the contour plot offers the most information and refers to the shape of a ridge associated with *θ* values along the diagonal from −3.00 to 2.50 for subjective well-being and 5.00 to −5.00 for positive affect (see Fig. 5). To simplify the results even more, we focused also on multidimensional item information function, based on marginal slopes, by keeping the value of the subjective well-being constant (e.g., *θ* = 0.00, *θ* = 4.00 and *θ* = −4.00) and leaving *θ* for positive affect free to vary between (e.g., −3 and 3). See Fig. 6, Fig. 7 for the details. In short, this item could not provide informative measurement about either the general subjective well-being factor or the positive affect subfactor for respondents who are either high on both factors or low on both factors.

Finally, we conducted Pearson correlations between the different scales, in order to test convergent and discriminant validity within the model. These correlations ranged between −0.57, *p* < .01 to .82, *p* < .01. Life Satisfaction (*r* = 0.61, *p* < .01) and harmony in life (*r* = 0.67, *p* < .01) were positively correlated with the positive affect. Conversely, negative affect was negatively correlated to life satisfaction (*r* = −0.50, *p* < .01) and harmony in life (*r* = −0.57, *p* < .01). Moreover, the positive affect and negative affect were negatively and correlated with each other (*r* = −0.45, *p* < .001). The largest correlation was that between life satisfaction and harmony in life (*r* = 0.82, *p* < .01). Thus, suggesting sufficient convergent and discriminant validity between the model's four different subfactors. These results agree with previous studies (e.g., Nima et al., 2020 ab).

## 4 Discussion

In the present study, we aimed to further validate our proposed tridimensional model of subjective well-being [15,38,39] by applying Bifactor-GR MIRT [50,54] We expected to simultaneously replicate our past findings with regard to the tridimensionality of subjective well-being in which we used separate techniques (i.e., CTT and UIRT) in two different samples.

At a general level each of the 30 items had a strong capacity to discriminate between respondents across all three dimensions of subjective well-being. The subjective well-being factor could explain 64 % of the common variance in the whole model, while 36 % was spread across the four constructs, hence, replicating findings using CTT [38] and suggesting that our data fit a multidimensional construct.^7^ Moreover, we found that with few exceptions (i.e., the positive affect items “Alert”, “Determined” and “Attentive”) the items measuring positive affect, life satisfaction, and harmony in life were better at discriminating among respondents and provided better information for locating individuals along their subjective well-being levels, than along levels of positive affect, life satisfaction, and harmony in life—we know this because the conditional and marginal slopes for the general subjective well-being latent factor were larger than the conditional and marginal slopes for positive affect, life satisfaction, and harmony in life. Conversely, most of the negative affect items, except for the items “Distressed” and “Irritable”, were better at discriminating among respondents and provided greater information for locating individuals along levels of negative affect than along levels of subjective well-being. Also in this line, the investigation of other parameters (e.g., ECV, IECV) strongly reflected the multidimensionality of subjective well-being in exactly the same way—at the item level, at the scale level, and at the model level (a) positive affect had a moderate strong tendency, life satisfaction had a strong tendency, and harmony in life had an extremely strong tendency for subjective well-being rather than their respective original concepts, (b) negative affect had a clear moderate strong tendency for its original concept rather than for subjective well-being, and (c) that across the analyses the same recurrent items were the exception to these coherent and recurrent results.

This set of results are in line with our expectations and past research using different techniques and samples [[16], [17], [18],^20^,^38^,^39^,^56^]. Moreover, since positive affect, life satisfaction, and harmony in life target positive life experiences in different planes of life, whereas negative affect target negative ones; this result also support the common notion of “bad is stronger than good” with a slight modification: “bad is distinct from good”. From a methodological perspective, it would be easy to just assume that the “individuality” of negative affect within the model has to do with response bias. Indeed, negative affect items could be seen as “opposite positive affect items” that also measure subjective well-being—that is, negative affect items are only positive affect items with reverse wording [57]. As the matter of fact, this notion is sometimes implicitly endorsed by researchers when they model subjective well-being as a composite subjective well-being standardized score in which a person's negative affect score is simply subtracted from the sum of her positive affect and life satisfaction scores [17]. If it is so, the response bias explanation is supported by past research showing that individuals make more mistakes when responding a 20-items instrument that includes reversed items, compared to when responding to the same instrument with 10 items posed in the same direction [58]. Indeed, the measure used here to operationalize the affective dimension, the Positive Affect Negative Affect schedule (PANAS), contains 20 items (10 positive affect, 10 negative affect) in random order.

Although the response bias explanation is plausible, we argue that negative affect's “individuality” within our proposed tridimensional model of subjective well-being has to do with negative affect and negative experiences as a concept and phenomena. First, there is extensive evidence that positive and negative experiences are best thought as two distinct and dissociable systems rather than opposite ends of a single continuum [59]. Indeed, positive and negative affect represent two general biobehavioral systems that lead to different motivational orientations. Positive affect is related to the *Behavioral Activation System* (BAS), which is sensitive to reward as well as approach motivation; while negative affect is related to the *Behavioral Inhibition System* (BIS), which is sensitive to signals of punishment as well as avoidance motivation [60,61]. Second, negative affect is more strongly genetically influenced than positive affect and it is more stable through life [25,62]. Third, from an evolutionary perspective, it has been argued that bias for negative stimuli has grown part of the toolbox of most animals, including humans, since it increases our chances of survival [[63], [64], [65]]—for example, many studies suggest that people process negative stimuli more elaborately than other stimuli [66,67]. Hence, items that measure negative affect cannot be seen as “reverse worded positive affect items”, because negative affect has different etiological and biopsychosocial basis, which makes it distinct and perhaps more independent than the other constructs within the tridimensional model of subjective well-being.

Most of the 30 items in the model were moderately to very good for discriminating between individuals with different levels of subjective well-being. Moreover, the examination of the multidimensional item difficulty parameters showed that the scales for positive affect, life satisfaction, and harmony in life were not able to measure high levels in these constructs. Therefore, we recommend the addition of items or the modification of current items that cover the experience of high levels of positive affect, life satisfaction, and harmony in life. Importantly, some items had very low discrimination values: the positive affect item “Alert” had a low discrimination value for subjective well-being, the life satisfaction item “I am satisfied with my life” had low discrimination value for life satisfaction, and all harmony in life items had low discrimination value for harmony in life. Regarding the positive affect item “Alert”, we have earlier argued [38,39] that besides the characteristic dimension of valence (positive and negative), affect has as a second characteristic dimension: activation/deactivation [[68], [69], [70]]. Hence, emotions can be vertically (from activation to deactivation) and horizontally (from negative to positive) organized as part of a circumplex [70]. In this circumplex, “Alert” is categorized on the highest point of the high activation dimension and at the lowest point of the positive dimension—thus, indicating that “Alert” has high arousal and low levels of pleasure as its core. In this context, past research suggest that happy people experience intensive positive emotions very rarely [[26], [27], [28]]. Ergo, the specific characteristic of “Alert” as a low pleasure/high activation affect might explain the problems highlighted using UIRT [39], CTT [38], and even here using MIRT. Thus, we recommend modification or exclusion of this item in order to address these issues. For instance, other instruments, like the Emotional Well-Being Scale [71], used to operationalize the affective dimension include items such as “Happy” and “Contended”, which might be better candidates because within the circumplex of emotions they are categorized as being high in pleasure, but moderately in activation/arousal [68].

However, some issues regarding specific items cannot be resolved by simply modifying or taken them out of the model. At first sight, it feels counterintuitive that the life satisfaction item “I am satisfied with my life” is better at measuring subjective well-being than at measuring life satisfaction as a specific construct. Also in this vein, the five items in the harmony in life measure, including “I am in harmony”, were better at measuring subjective well-being than harmony in life. Any modification to such items would require to re-word around each of the concepts, but which items can conceptualize satisfaction with a self-imposed ideal better than “I am satisfied with my life” and conceptualize the sense of harmony and balance better than “I am in harmony”? For instance, empirical research using both qualitative and quantitative methodology across individualistic and collectivistic cultures (e.g., Australia, Croatia, Germany, Italy, Portugal, Spain, and South Africa) shows that lay people's definitions of happiness, at both the individual and social levels, can be primarily organized (about 29 % of the definitions) in a “balance/harmony” theme [37]. This seminal cross-cultural work also shows that, out of 13 themes used to categorize lay people's definitions of happiness, the second theme is “satisfaction” (about 17 % of the definitions), and the third theme is “positive emotions” (about 14 % of the definitions); while negative emotions are only represented in a theme labelled “no negative feelings” (about 5 % of the definitions) placing as number *seven* out of the 13 themes [37]—that is, about 65 % of lay people's definitions of happiness had to do with harmony, satisfaction, positive emotions, and lack of negative emotions, in that specific incremental order. In other words, the fact that these items (e.g., “I am satisfied with my life” and “I am in harmony”) have a larger effect on subjective well-being than on its respective concept, is in line with how people think about happiness. In addition, the magnitude of how each of the constructs in our model relate to subjective well-being as a whole seems to mirror how people define happiness: first and foremost as harmony in life, which is almost one with subjective well-being; second satisfaction with life, which had a strong tendency towards the whole; third as positive emotions, which had a moderate strong tendency towards the whole; and last negative emotions, which were a more independent part within the whole.

Last but not the least, the results revealed that the probability of selecting a certain response to the 30 items in the model is conditionally associated with the various combinations of the four constructs in the tridimensional model of subjective well-being [50,54]. Respondents that reported high levels of subjective well-being, but low levels in one of the specific constructs, had the tendency to choose a high-level response to each of the other items measuring that specific construct. Conversely, respondents that reported low levels of subjective well-being in conjunction with high levels in one specific construct, tended to choose a low-level response to each of the other items measuring that specific construct. Thus, reflecting the compensatory nature of subjective well-being as a tridimensional construct or complex adaptive system, where a person's high levels in, for example, harmony in life compensate for her low levels of life satisfaction and vice versa [42,50,54]. From a methodological perspective, the nature of subjective well-being as a multidimensional complex adaptive system [15] implies that advanced statistical techniques, as the ones used here, are required to measure people's subjective experiences of well-being. From a theoretical and empirical perspective, measuring each dimension separately is not an accurate way to understand people's subjective well-being, it rather needs to be measured as a tridimensional bifactor structure. Likewise, subjective well-being cannot be measured as a single structure isolated from its three dimensions and four constructs. As the matter of fact our findings are similar to recent ground-breaking research showing that the seven most common instruments used to measure depression encompassing 52 disparate symptoms [72], are actually not unidimensional in nature, and that the sum-scores approach does not reflect the underlying construct of depression [22]—there is, for example, a myriad of emotion items that could be included [68,69] and we have shown that the sum-scores approach is not appropriate for measuring subjective well-being [38,39].

### 4.1 Strengths, limitations and implications for current research

To the best of our knowledge, no study has taken advantage of MIRT to examine the psychometric properties and dimensionality of subjective well-being, its three dimensions (i.e., affective, cognitive, and social/behavioral), and its four constructs (i.e., positive affect, negative affect, life satisfaction, and harmony in life). We argue that Bifactor-GR MIRT is the recommended technique to use, since it enables to model and develop an intuitive understanding and explanation of the interactions regarding the vectors that represent underlying multiple dimensions. With MIRT we can test the structures of the dimensions by treating them as different latent dimensions in order to assess the multidimensionality of the model in which the items reflect the different evaluation processes people make regarding their affective experience and apprehension of life satisfaction and harmony in life.

However, the residuals in the model were mostly significantly correlated, which also indicates that the data had tendency to a multifaceted construct, rather than the multidimensionality of a bifactor model. Thus, we might need to apply more complex models than bifactor model to deal with the potential level of the violation of local independence, which is probably related to the construct irrelevant variance. Future studies could test other MIRT models as proposed solutions to cope with the potential violation of the assumption of local item independence. The Testlet Model and Two-Tier Model, for example, may be used as a constrained version of a Bifactor MIRT model [73], which includes a general single latent factor (primary factor) and a set of specific latent subfactors and in which the variances of the testlet effects (specific factor effects) are freely estimated [74]. Moreover, the specific factor loadings (item slop parameters) within each testlets and the general loadings are constrained to be equal. Two-Tier Model includes general latent factors (primary tier) and a set of specific latent subfactor (secondary tier). In Two-Tier Model, specific factors are assumed to be uncorrelated and have a fixed variance of 1, while the general factors could covary with other general factors. These models (Testlet and Two-Tier Model) could be applied to valuate and eliminate the impact of local item dependence on the reliability and the validity of inferences from the scales. Another technique that might be useful is Latent Profile Analyses, which discerns subgroups of individuals with different subjective well-being profiles [75]. In short, Latent Profile Analysis helps researchers to gain insights into “hidden” psychological interactions to generate profiles and is a kind of person-centered modeling.

Even though we have argued for harmony in life and life satisfaction being at the core of subjective well-being, the items in these scales might consist of words that do not directly address, for example *balance* or *adaptation*, words that lay at the core of harmony in life as a concept [35]. In other words, it is plausible that such straightforward questions (e.g., “I am satisfied with my life”, “I am in harmony”) get lost in translation, because they ask about the concept of satisfaction and harmony directly; concepts that are extremely abstract that individuals lack a specific or defined mental schema for [38,39]. This makes it more difficult for participants to discriminate between the concepts and for researchers to understand the responses. We agree that understanding that lay people define happiness primarily as harmony and secondly as satisfaction is important, but the question is then if our results mirror harmony and satisfaction as the core of a multidimensional structure or only reflect how poorly the instruments are at measuring the specific dimensions of subjective well-being. If modification of items is the path to walk, we might need to consider that each construct needs to be operationalized using all the properties of the concept (i.e., feelings, cognitions, and behaviors) rather than the concept itself—that is, a holographic structure in which every part resembles the whole; for example, which emotions are part of the sense of harmony, what are the thoughts of being in harmony, and what are the behaviors representing harmony?, see also [7,76]. Without considering how people express their well-being and past relevant research beyond a specific field, we risk ending up with “quick and dirty measures” that lack a comprehensive theory [77] and suffer of “jingle-jangle” fallacy [78]. In this context, a scientific model represents phenomena (in this case subjective well-being) in a logical but simplified way [[79], [80], [81], [82], [83], [84]]. As such, the tridimensional model of subjective well-being should be interpreted and used with caution and therefore it is recommendable that future studies also validate if each of the dimensions are affective, cognitive, and behavioral in nature.

Another limitation is that we collected the data through Amazon's Mechanical Turk, which is as a useful tool that is still widely applied for data collection in the social and psychological fields. However, there are recent indications of declining data quality [85]. Although this has happened after our data collection, our approach to “cleaning” up the data yielded a large amount of data being excluded from the final analysis. We argue that this was important to avoid low data quality having a negative impact on our study results and conclusions. However, we had to drop a lot of data, which at the end might have some repercussions on how heterogenous our data is. Moreover, for investigating convergent and discriminant validity, we only used the subscales within the tridimensional subjective well-being model. Thus, future studies should test the model's relationship to personality—which is probably the strongest single determinant of people's well-being [7].

All that being said, one of the major concerns in past subjective well-being research was how to conceptualize and measure its distinct dimensions. Most researchers now agree that the affective dimension is best conceptualized as how (in)frequent people experience specific emotions rather than how intensively [[23], [24], [25], [26], [27],[29], [30], [31],^86^]. To the best of our knowledge, however, there is no consensus on which specific emotions should be included to operationalize the emotions that best represent the happy life.^8^ Currently, the choice seems relatively arbitrary, for example, by convenience (e.g., fewer items when time is an issue) or based on which scale is the most “acceptable” one. Since we here evaluate subjective well-being at three levels of analyses (i.e., item, scale, and general factor) simultaneously, our results shade some new light to this relatively untouched but important issue.

First, frequently experiencing feeling distressed and irritated were the only two negative emotions and feelings, out of the 10 measured by the PANAS, that better measured subjective well-being as a whole and that at the same time discriminated between individuals with different levels of negative affect. Interestingly, distress and irritable are emotions located in the active-displeasure dimension of the circumplex of emotions but they are not as high in the activation dimension as the other negative emotions in the PANAS. They are rather between activation and deactivation [68,87]—that is about 45° in the quadrant unpleasant-activation of the circumplex and therefore best understood as a blend of displeasure and arousal rather than just a plain unpleasant emotion or a plain high arousal emotion [70]. Also is this vein, “Alert” was the only item in the positive affect scale of the PANAS that failed to measure subjective well-being as a general factor. “Alert” is indeed a high activation positive emotion at the top of the pleasant-activation quadrant [68,87]. As pointed out by others [[68], [69], [70],^87^], semantic and lexical studies suggest that there is a large range of words that represent our emotional experience, for example, between feeling distressed and feeling depressed. Hence, based on our results, we can conclude that subjective well-being researchers might need to stay away from most of the negative emotions measured by the PANAS (i.e., all but “Distressed” and “Irritable”) and use other measures that include plain unpleasant emotions with low arousal or those that are a blend of displeasure and arousal. Nevertheless, the problem might as well be that these self-report scales pre-impose specific feelings and emotions to measure people's affective experience, which at the end is not an optimal way of measuring the subjective experience of how people evaluate the emotional reactions derived from their own nervous system [88]. Therefore, other methods that allow people to report which positive and negative emotions they frequently experience need to be explored and developed [88,89]. See also https://replicationindex.com/2022/05/28/hedonimus/.

Second, based on the present results, negative affect seems to be a separate dimension rather than part of the whole, at least as measured by the PANAS. This is an extremely important issue since the consensus or rather the most convincing evidence to date, is that subjective well-being should be operationalized as a general factor (i.e., higher order latent factor) to which the variance in each subjective well-being dimension is attributable to. This approach accounts for both a common source variance and a unique source variance in each dimension [[16], [17], [18], [19], [20],^56^]. This unity of subjective well-being is then not justified if negative affect contributes more to its own variance rather than to the general factor. Thus, the question is if negative affect should be removed as one of the constructs of the affective dimension? Even though it is too early to suggest something this radical, our [38,39] and others' current research [[16], [17], [18], [19], [20], [21]] have (a) confirmed the originally theorized dual dimensionality of subjective well-being [2], (b) replicated and reinforced, using new and advanced statistical tools, the methodological principle of subjective well-being as multi-dimensional rather than an unidimensional single construct [[17], [18], [19], [20],^56^], (c) put forward quantitative evidence, for the operationalization of subjective well-being, that mirrors observations by other researcher about how ancient civilizations saw the ability to maintain balance and serenity in both happy and challenging times as part of the happy life [34,37] and that also (d) mirrors qualitative and quantitative studies across individualistic and collectivistic cultures showing that lay people primarily define happiness as psychological balance and harmony and life but as satisfaction and the presence of emotions, specially negative emotions, only as subordinate definitions [37]. Ergo, further verification of negative emotions’ lack of contribution to a subjective well-being general factor will strengthen the evidence supporting the removal of negative emotions, especially high activation ones, when operationalizing and measuring subjective well-being.

### 4.2 Conclusion and final remarks

Many past studies have addressed subjective well-being and its two original dimensions (i.e., affect and life satisfaction). Our results suggest the addition of a third dimension and reflected the true multidimensional nature of subjective well-being. In addition, people's experience of high levels in one subjective well-being dimension compensates for their experience of low levels in the other dimensions and vice versa. The knowledge and awareness of this compensatory nature between dimensions within subjective well-being is important for the conceptualization, operationalization, and understanding of the cognitive mechanisms and processes behind people's evaluations and judgements of their subjective well-being and each of its constructs. Consequentially, due to this interconnection, interventions aiming to promote well-being, need to be ternary in nature, that is, promoting aspects of being that not only increase positive emotions, but also increase life satisfaction and harmony in life and decrease negative emotions, see for example [[90], [91], [92], [93], [94], [95], [96], [97], [98]]. By simultaneously influencing emotions, cognitions, and behaviors, or factors that lay at the core and regulate these dimensions, such as character; [7,[99], [100], [101], [102], [103], [104], [105], [106], [107], [108]], we might be able to help people to develop sustainable happiness, resilience, virtues, and an outlook of unity to adapt to the current and future challenges of the 21st century [91,92,101,108].

In addition, for clinicians it is important to be aware that the dimensions are interdependent to each other and that the dynamics of these relationships are probably distinct for each dimension [22,72,109]. That is, the awareness of this processes might help clinicians and practitioners to understand why some people experience life as meaningless or are not fully happy, despite reporting being satisfied with life or being close to their ideal self—perhaps they experience positive emotions very infrequently and lack the sense of balance and harmony in life. In this context, to understand people's improvement and the process within subjective well-being and its three dimensions, clinicians and practitioners should be aware that complex statistics are needed if the dynamics within the construct are fully considered. Hence, tracking patients or clients' improvement needs to be done using either technology with imbedded statistical solutions or in collaboration with statisticians and researchers.

Finally, the tridimensional model tested here has parallels with the biopsychosocial model of health and the definition of health by the World Health Organization—that is, the notion that health is, besides the absence of disease, also a state of physical, mental, and social well-being, see also [7,[110], [111], [112]]. In this context, the affective dimension consists of evaluations of one's emotions, which are derived from our biological nervous system and temperament traits with a strong genetic factor and therefore relatively stable through life [7,52,113]; the cognitive dimension consists of own judgements of a psychological self-imposed ideal [4]; whereas harmony in life consist of the apprehension of one's sense of balance and capacity to behave and adapt with both acceptance and flexibility to circumstances within and outside the self. In short, subjective well-being is the holistic apprehension or our attitude towards life in these three dimensions—how we feel, how we think, and how we behave [15], that is, the A (ffect), B (ehavior), C (ognition) of happiness.

*“The whole is greater than the sum of its parts.”*

Aristotle

*“I … a universe of atoms, an atom in the universe.”*

Richard P. Feyman

## Data availability statement

Data associated with this study has not been deposited into a publicly available repository, but it will be made available on request.

## CRediT authorship contribution statement

**Ali Al Nima:** Writing – original draft, Methodology, Investigation, Formal analysis, Data curation. **Danilo Garcia:** Writing – review & editing, Writing – original draft, Supervision, Methodology, Data curation. **Sverker Sikström:** Writing – review & editing. **Kevin M. Cloninger:** Writing – review & editing, Conceptualization.

## Declaration of competing interest

The authors declare that they have no known competing financial interests or personal relationships that could have appeared to influence the work reported in this paper.

## Footnotes

1 The quantification of words based on their semantic meaning in relation to words in natural language [89,[114], [115], [116], [117], [118], [119], [120], [121], [122], [123], [124]].

2 Amazon's Mechanical Turk (http://www.mturk.com/mturk/welcome) gives researchers the possibility to recruit online participants (i.e., workers) for answering surveys and good data quality [85,125,126].

3 Discrimination parameters' values between 0.01 and 0.34 are considered as very low; between 0.34 and 0.64 as low; between 0.65 and 1.34 as moderate; between 1.35 and 1.69 as high; and 1.70 and above as very high [44,127].

4 The explained common variance (ECV) represents the sources of item variance that depend to the single general factor and subfactors. More specifically, ECV can help to assess the dimensionality of the model and to clarify the interpretation of the relationship between the general factor (ECVg) and its subfactors (ECVs). In short, ECV_g_ is a statistical reliability index in a bifactor analysis that refers to the proportion of common variance in the observed scores across all items explained by the general factor, ECV_s_ refers to the proportion of common variance in the observed scores across all items explained by the subfactors. Hence, the sum of ECVg and ECVs values is always equal to 1 [128,129].

5 The explained common variance for the general factor at the item level (IECVg) is as a statistical reliability index in a bifactor analysis that indicates how much of the common reliable variance at the item level is explained by the general factor; while the explained common variance for the subfactors at the item level (IECVs) refers to how much of the common variance at the item level is explained by a subfactor. The sum of IECVg and IECVs values is always equal to 1 [55,130,131].

6 Multidimensional item information surface reflects how much information each item can provide and reflect the area of the continuous latent variables in the multidimensional space in which measurement of the respondent parameters is most precise. Importantly, the concept of information in IRT is inversely related to error.

7 According to previous studies an ECV below 70 % indicates multidimensionality (i.e., it can be broken into multiple scales), whereas an ECV above 90 % indicates unidimensionality [130,132].

8 For a substantial critical review on this topic, please see: https://replicationindex.com/2022/05/28/hedonimus/. See also [84,133].
