# Supplementary material for: The ABC of happiness: Validation of the tridimensional model of subjective well-being (affect, cognition, and behavior) using Bifactor Polytomous Multidimensional Item Response Theory
Source: Heliyon. 2024 Jan 12;10(2):e24386. doi: 10.1016/j.heliyon.2024.e24386 (PMC10831611; doi:10.1016/j.heliyon.2024.e24386)
Supplement: Multimedia component 1 [file mmc1.pdf]

**SUPPLEMENTARY MATERIAL**

# The ABC of Happiness: Validation of the Tridimensional Model of Subjective Well-Being (Affect, Cognition, and Behavior) using Bifactor Polytomous Multidimensional Item Response Theory

Ali Al Nima[1, 2]*, Danilo Garcia[1, 2, 3, 4, 5]*, Sverker Sikström[2, 5], Kevin M. Cloninger[6, 7, 8]

[1]Department of Psychology, University of Gothenburg, Gothenburg, Sweden

[2]Promotion of Health and Innovation Lab (PHI), International Network for Well-Being, Sweden

[3]Department of Behavioral Sciences and Learning, Linköping University, Linköping, Sweden

[4]Centre for Ethics, Law and Mental Health (CELAM), University of Gothenburg, Sweden

[5]Department of Psychology, Lund University, Lund, Sweden

[6]College for Public Health and Justice, Saint Louis University, St. Louis, Missouri, USA

[7]Anthropedia Foundation, St. Louis, Missouri, USA

[8]Promotion of Health and Innovation Lab (PHI), International Network for Well-Being, USA

*Correspondence regarding this article should be addressed to A. A. Nima, alinor_1979@yahoo.co.uk, or D. Garcia, danilo.garcia@icloud.com.

**Methodological Assumptions for Conducting Multidimensional Item Response Theory (MIRT)**

To apply IRT models (both UIRT and MIRT), there are some basic assumptions: unidimensionality (1), local independence (2), and monotonicity (3) (Bonifay, 2020; Toland, Sulis, Giambona, Porcu, & Campbell, 2017). Unidimensionality (1) refers to where each item loads on only a single continuous underlying factor to explain the collection of item response pattern in a given set of items or measurement. This assumption can be tested using, for example, factor analysis. When this assumption is violated, the results obtained using UIRT could lead to misleading and biased parameter estimates. Multidimensionality exists when some or all items, in a test or construct, load on more than a single continuous underlying trait to explain the item response patterns. Multidimensionality can be classified as within-item and between-item multidimensionality (Paek & Cole, 2018). In the between-item multidimensionality, each item in a given measure are modelled by one of several latent dimensions. While within-item multidimensionality occurs when some or all items within the set of tests belong to more than one single latent dimension or trait. Local independence (2) refers to the independence between how individuals respond to one item and how they respond to all the other items in the scale, given the individual's score in the latent variable. This is usually tested by verifying that the residuals for each item are not significantly correlated to the residuals of any other item in the same scale. Last but not the least, monotonicity (3) refers to item characteristics reflecting the functional association between the person's unobserved trait score and response pattern to the item. In other words, IRT models assume that the levels of the person's latent trait increase, as a monotonical function, as the probability to choosing the answer in each item that represents the participants actual level of the trait increases (Nima et al., 2020a). This relationship between respondents' observable item answers and their set of unobserved traits could be violated when respondents with lower levels of the latent traits have

higher probabilities of an observable correct response (i.e., answering higher values for items when the person is actually low in, for example, subjective well-being) than respondents with higher levels of the latent traits. Such types of violations of the monotonicity assumption, sometimes suggest that an item that is reverse-worded was not reverse-coded before data analysis (Bonifay, 2020). Typically, the monotonicity function can be evaluated by using, for example, model-data fit statistics **(**Paek & Cole, 2018). Next, we describe the procedure applied when we tested each one if these three assumptions in our data.

Regarding the assumption of local independence, we used *LD-X2* (Chen & Thissen, 1997) as the index for local independence and to calculate *Cramer V* coefficient. The *Cramer V* coefficient has a range value from -1 to +1, and it is an extension of the *phi* coefficient. In the present study, the residuals (i.e., the differences between the observed scores and the predicted scores) of virtually each paired correlation were significant (See Table S1a and S1b in the Supplementary Material for the details). Moreover, as suggested by Yen (1984) we used the *Q3* statistic, which refers to the correlation of residuals for each item pair and therefore ranges from -1 to +1, to measures the local dependence violation (i.e., a value that is larger than the absolute value, that is, > 0.2236). Our data showed a pattern of absolute values larger than 0.2236: 11 values among item pairs within negative affect (NA), 3 values among item pairs within positive affect (PA), 1 value among item pairs within life satisfaction (SWLS) and 1 value among item pairs within harmony in life (HILS). Importantly, the item "Scared" (with "Distressed", "Afraid", "Ashamed" and "Nervous") and "Irritable" (with "Distressed", "Upset" and "Hostile") showed absolute values larger than 0.2236 with several items within the negative affect construct (See Table S2 in the Supplementary Material for the details). Thus, the unmodeled covariations between item pairs was especially true for NA. Nevertheless, it is important to take into consideration that most of the values larger than 0.2236 are close to 0.2236 and also the large number of correlations—435 correlations of residuals for each item

pair regarding 30 items in the matrix (30x30). In other words, although the result regarding local independence using different indices (*LD-X2*, *Cramer V* coefficient, and *Q3* statistic) does not perfectly reflect the local independence between items pairs, Bifactor-GR MIRT could totally explain most of the item response patterns or covariations within our suggested model, in which subjective well-being is a general dimension with four separate specific dimensions (i.e., positive affect, negative affect, life satisfaction, and harmony in life). What is even more, the Bifactor-GR MIRT could largely improve the modelling regarding to item response covariates compared with our previous study using UIRT (Nima et al., 2020b), in which the residuals of almost each paired correlation for all four dimensions (i.e., positive affect, negative affect, life satisfaction, and harmony in life) were significant, the data showed a tendency for locally dependency for most of the items and for multidimensionality in general.

Regarding the assumption of monotonicity, we tested model-data fit statistic for both item level and global model. To test the fit of the model to each item, we used the Orlando-Thissen-Bjorner item fit $S\text{-}\chi 2$ statistic test and found that 8 items were adequately fit (i.e., nonsignificant values), while 22 items were statistically significant. Nevertheless, this test is sensitive to sample size, test length, and multiple comparisons, which might yield larger values, thus, a larger chance of being significant (i.e., Type I error). Thus, these 22 items were probably falsely identified as mis-fitting. Therefore, to further determine the absolute fit of the model to each item, we applied the root mean square error of approximation based on the $S\text{-}\chi 2$ statistic (*RMSEA* $S\text{-}\chi 2$), and found that the largest value of *RMSEA* $S\text{-}\chi 2$ in our data was .07, thus, indicating an adequate item-level model-data fit for all items—the traditional cut-offs for *RMSEA* tend to be $\leq 0.08$. Additionally, we used the Benjamini-Hochberg (Benjamini & Hochberg, 1995) criterion for *p*-value adjustment. The majority of items were adequately fit but six items were still significant after correction (See Table S3 in the Supplementary Material for the details). In order to determine the global model-data fit, we used various fit indices. The

results showed that the limited information goodness-of-fit test statistic $M_2$ value was significant ($M_2$ = 483.00, *df* = 265, $p < .001$). Since, larger samples lead to a larger $M_2$ values (i.e., a larger likelihood of being significant), we focused on all other fit indices. All these other fit indices indicated that the model fit was acceptable: the Tucker-Lewis Index (*TLI*) was .94, the comparative fit *index* (*CFI*) was .95, the Standardized Root Mean Square Residual (*SRMSR*) was .04 and the *RMSEA* fit statistic was .04.

## References


Bonifay, W. (2020). *Multidimensional Item Response Theory.* New York: Sage.

Nima, A. A., Cloninger, K. M., Persson, B. N., Sikström, S., & Garcia, D. (2020a). Validation of Subjective Well-Being Measures Using Item Response Theory. Frontiers in Psychology 10:3036. doi: 10.3389/fpsyg.2019.03036.

Paek, I., & Cole, K. (2019). *Using R for Item Response Theory Model Applications* (1st ed.). Routledge. https://doi.org/10.4324/9781351008167

Toland, M. D., Sulis, I., Giambona, F., Porcu, M., & Campbell, J. M. (2017). Introduction to bifactor polytomous item response theory analysis. Journal of School Psychology, 60 (Supplement C), 41-63. doi:https://doi.org/10.1016/j.jsp.2016.11.001

Table S1a: LD-X2 values and the standardized residuals (Cramer's V Coefficient) among 30 items in all measures in the study. (*n* = 435).

| Items | 1 | 2 | 3 | 4 | 5 | 6 | 7 | 8 | 9 | 10 | 11 | 12 | 13 | 14 | 15 | 16 | 17 | 18 | 19 | 20 | 21 | 22 | 23 | 24 | 25 | 26 | 27 | 28 | 29 | 30 |
|---|---|---|---|---|---|---|---|---|---|---|---|---|---|---|---|---|---|---|---|---|---|---|---|---|---|---|---|---|---|---|
| SWLS01 | NA | 0.24 | -0.23 | -0.26 | -0.21 | 0.19 | -0.14 | 0.14 | -0.17 | -0.15 | -0.17 | -0.13 | -0.20 | -0.15 | -0.20 | -0.23 | -0.18 | -0.19 | -0.17 | -0.15 | -0.12 | -0.14 | 0.18 | 0.14 | 0.16 | -0.12 | 0.13 | -0.13 | -0.11 | 0.16 |
| SWLS02 | 149.02 | NA | -0.33 | -0.23 | -0.23 | -0.13 | -0.19 | -0.16 | -0.17 | -0.16 | -0.16 | -0.11 | -0.16 | -0.14 | -0.15 | -0.13 | -0.14 | -0.15 | 0.15 | -0.12 | 0.18 | 0.17 | 0.16 | 0.15 | 0.16 | 0.13 | 0.13 | 0.13 | -0.12 | 0.16 |
| SWLS03 | 134.09 | 275.87 | NA | 0.19 | -0.20 | -0.18 | -0.20 | -0.20 | 0.16 | -0.15 | -0.15 | 0.14 | -0.21 | -0.16 | -0.15 | -0.14 | -0.16 | -0.16 | -0.16 | -0.15 | -0.14 | -0.21 | 0.17 | 0.14 | 0.15 | -0.14 | -0.16 | -0.14 | -0.14 | 0.19 |
| SWLS04 | 179.91 | 143.52 | 93.35 | NA | 0.18 | -0.24 | -0.18 | -0.16 | 0.18 | -0.15 | -0.14 | -0.13 | -0.16 | 0.14 | -0.15 | -0.15 | -0.14 | -0.18 | -0.19 | -0.15 | -0.20 | -0.24 | -0.14 | -0.14 | 0.16 | -0.14 | -0.13 | -0.12 | -0.14 | -0.17 |
| SWLS05 | 110.03 | 136.01 | 108.66 | 86.49 | NA | -0.18 | -0.17 | -0.18 | -0.19 | -0.15 | -0.17 | -0.13 | -0.17 | -0.13 | -0.16 | -0.15 | -0.15 | -0.12 | 0.15 | -0.15 | -0.12 | -0.13 | -0.16 | -0.13 | -0.14 | -0.14 | -0.14 | -0.12 | -0.13 | -0.15 |
| HILS01 | 92.28 | 43.45 | 86.02 | 151.75 | 87.93 | NA | -0.43 | -0.35 | -0.16 | -0.17 | -0.20 | -0.15 | -0.20 | -0.17 | 0.20 | 0.16 | 0.20 | -0.23 | -0.16 | -0.14 | -0.16 | -0.26 | 0.15 | 0.21 | 0.15 | -0.17 | 0.15 | -0.15 | 0.17 | -0.18 |
| HILS02 | 54.29 | 94.20 | 100.18 | 84.22 | 71.69 | 472.40 | NA | -0.69 | -0.16 | -0.16 | -0.16 | -0.15 | -0.23 | -0.17 | -0.18 | -0.16 | -0.19 | -0.19 | -0.19 | -0.16 | -0.19 | -0.16 | 0.15 | 0.20 | -0.15 | -0.17 | 0.14 | 0.15 | 0.16 | -0.16 |
| HILS03 | 53.22 | 65.98 | 106.51 | 66.44 | 80.64 | 311.59 | 1228.1 | NA | -0.16 | -0.15 | -0.16 | -0.16 | -0.18 | -0.17 | 0.18 | 0.16 | 0.19 | -0.17 | -0.17 | -0.16 | 0.15 | 0.25 | 0.18 | 0.18 | 0.16 | -0.16 | 0.18 | 0.18 | 0.15 | 0.19 |
| HILS04 | 78.29 | 72.19 | 67.86 | 88.50 | 89.66 | 68.72 | 70.35 | 64.44 | NA | 0.22 | -0.13 | -0.16 | -0.15 | 0.14 | -0.11 | 0.13 | 0.16 | -0.16 | -0.15 | -0.13 | 0.17 | 0.15 | 0.16 | 0.16 | 0.14 | 0.16 | 0.13 | -0.15 | 0.15 | 0.20 |
| HILS05 | 59.87 | 62.69 | 58.71 | 55.96 | 56.22 | 77.21 | 69.02 | 59.30 | 126.30 | NA | 0.12 | -0.11 | 0.15 | 0.15 | 0.12 | 0.12 | 0.15 | -0.20 | 0.15 | -0.14 | -0.12 | -0.14 | -0.13 | 0.13 | 0.15 | -0.14 | -0.12 | -0.13 | 0.12 | 0.13 |
| Interested | 49.61 | 45.03 | 38.01 | 31.82 | 50.34 | 67.24 | 46.12 | 43.34 | 30.76 | 25.06 | NA | 0.09 | -0.20 | 0.18 | -0.10 | 0.14 | 0.15 | -0.14 | -0.13 | -0.09 | 0.15 | 0.12 | 0.13 | 0.11 | 0.11 | 0.13 | 0.13 | 0.15 | 0.15 | 0.13 |
| Enthusiastic | 29.85 | 22.77 | 31.50 | 28.22 | 30.99 | 38.24 | 37.52 | 43.07 | 43.08 | 21.65 | 15.54 | NA | -0.23 | -0.21 | -0.16 | -0.17 | -0.15 | -0.15 | 0.15 | -0.15 | -0.13 | 0.13 | 0.11 | -0.10 | -0.12 | 0.10 | 0.10 | -0.08 | -0.10 | -0.12 |
| Proud | 66.18 | 42.15 | 73.12 | 42.38 | 49.78 | 67.85 | 89.38 | 57.96 | 38.67 | 37.65 | 70.69 | 94.55 | NA | -0.16 | 0.13 | -0.14 | -0.19 | -0.17 | 0.15 | 0.15 | -0.15 | -0.14 | -0.12 | -0.12 | -0.12 | -0.10 | -0.10 | -0.12 | -0.09 | -0.11 |
| Alert | 39.24 | 32.36 | 41.66 | 34.81 | 27.04 | 52.87 | 48.53 | 51.07 | 34.46 | 40.06 | 58.41 | 76.71 | 45.13 | NA | -0.14 | -0.12 | 0.25 | -0.20 | -0.16 | -0.14 | 0.15 | -0.15 | 0.17 | -0.14 | 0.13 | 0.18 | 0.14 | -0.11 | -0.13 | 0.14 |
| Inspired | 71.87 | 38.77 | 37.29 | 37.04 | 43.06 | 66.98 | 58.41 | 54.99 | 21.86 | 24.12 | 18.97 | 46.59 | 29.69 | 33.91 | NA | -0.16 | -0.19 | -0.16 | 0.15 | 0.13 | -0.07 | -0.12 | 0.12 | -0.10 | 0.10 | 0.10 | -0.12 | 0.11 | -0.10 | -0.12 |
| Determined | 91.03 | 27.14 | 34.34 | 37.21 | 41.05 | 46.49 | 44.29 | 46.32 | 29.74 | 26.85 | 34.61 | 52.63 | 33.34 | 25.91 | 44.64 | NA | 0.17 | -0.14 | -0.15 | 0.16 | -0.12 | 0.11 | 0.14 | 0.10 | 0.10 | 0.13 | 0.10 | 0.09 | 0.12 | -0.14 |
| Attentive | 54.76 | 33.76 | 42.23 | 35.61 | 37.15 | 69.50 | 59.57 | 63.48 | 46.34 | 41.47 | 38.86 | 38.76 | 59.58 | 111.97 | 64.13 | 50.29 | NA | -0.17 | -0.16 | -0.11 | 0.12 | 0.11 | 0.15 | 0.11 | 0.13 | 0.16 | 0.15 | 0.09 | 0.15 | 0.10 |
| Active | 60.65 | 38.61 | 46.38 | 56.72 | 25.32 | 90.35 | 63.31 | 52.75 | 42.31 | 69.37 | 34.72 | 39.04 | 48.91 | 71.22 | 45.74 | 33.94 | 48.55 | NA | -0.13 | -0.13 | 0.10 | 0.10 | 0.10 | -0.11 | 0.11 | 0.10 | 0.11 | 0.09 | -0.11 | 0.10 |
| Excited | 47.18 | 40.95 | 46.67 | 64.81 | 40.51 | 45.07 | 60.87 | 49.78 | 39.93 | 37.91 | 28.51 | 38.42 | 41.37 | 41.67 | 36.99 | 37.74 | 42.19 | 31.08 | NA | -0.16 | -0.16 | -0.19 | -0.13 | -0.13 | -0.13 | -0.10 | -0.12 | -0.13 | -0.14 | -0.14 |
| Strong | 36.74 | 25.44 | 38.21 | 38.10 | 38.35 | 34.16 | 42.31 | 42.31 | 28.56 | 34.18 | 14.85 | 36.79 | 41.22 | 35.99 | 30.83 | 43.41 | 21.84 | 27.31 | 41.71 | NA | -0.13 | 0.13 | 0.11 | -0.11 | 0.08 | 0.08 | 0.08 | 0.09 | 0.10 | -0.08 |
| RecodedDistressed | 25.35 | 56.22 | 31.98 | 67.81 | 25.88 | 46.29 | 63.85 | 37.50 | 52.56 | 26.43 | 37.21 | 27.81 | 37.78 | 36.33 | 8.45 | 26.06 | 23.10 | 15.55 | 44.02 | 28.22 | NA | 0.27 | -0.10 | -0.15 | -0.12 | 0.11 | -0.12 | -0.12 | -0.12 | -0.12 |
| RecodedUpset | 34.04 | 49.41 | 73.64 | 103.29 | 30.76 | 121.34 | 42.58 | 112.16 | 39.42 | 32.15 | 25.46 | 29.50 | 36.27 | 37.95 | 23.85 | 21.68 | 22.19 | 15.75 | 65.47 | 27.26 | 126.92 | NA | 0.12 | -0.13 | -0.12 | 0.11 | -0.23 | -0.24 | -0.11 | -0.24 |
| RecodedGuilty | 53.30 | 44.51 | 48.41 | 35.51 | 41.67 | 39.65 | 38.91 | 57.20 | 45.60 | 28.66 | 27.38 | 20.42 | 24.43 | 47.78 | 23.92 | 34.49 | 38.34 | 16.77 | 28.65 | 21.39 | 16.77 | 24.19 | NA | -0.08 | -0.11 | -0.11 | 0.19 | -0.10 | -0.13 | -0.12 |
| RecodeAfraid | 31.62 | 37.98 | 36.18 | 31.74 | 30.48 | 75.86 | 68.60 | 58.27 | 46.33 | 27.35 | 20.25 | 17.87 | 25.85 | 32.62 | 16.74 | 17.46 | 22.12 | 20.14 | 30.16 | 22.01 | 39.16 | 27.71 | 11.13 | NA | -0.12 | -0.16 | -0.13 | -0.10 | -0.11 | 0.14 |
| RecodeHostile | 41.87 | 46.74 | 37.16 | 45.61 | 34.30 | 41.33 | 40.19 | 42.66 | 31.90 | 37.16 | 19.01 | 23.63 | 23.29 | 27.07 | 18.77 | 17.90 | 27.95 | 19.77 | 28.39 | 10.48 | 23.08 | 24.46 | 19.21 | 26.91 | NA | 0.14 | -0.10 | -0.12 | -0.10 | -0.12 |
| RecodeIrritable | 25.61 | 27.71 | 33.79 | 35.94 | 32.44 | 52.13 | 51.86 | 42.66 | 42.07 | 32.71 | 28.32 | 16.22 | 18.80 | 53.14 | 18.40 | 29.69 | 47.00 | 17.20 | 16.80 | 10.58 | 20.46 | 20.55 | 20.62 | 42.72 | 31.56 | NA | -0.15 | -0.13 | 0.13 | -0.21 |
| RecodeAshamed | 29.01 | 30.66 | 45.69 | 29.34 | 35.15 | 39.19 | 36.27 | 54.00 | 28.90 | 24.90 | 28.89 | 16.73 | 15.58 | 34.49 | 23.93 | 17.05 | 38.93 | 19.22 | 24.67 | 10.66 | 26.30 | 95.68 | 59.58 | 30.28 | 18.03 | 37.16 | NA | -0.07 | -0.10 | -0.11 |
| RecodeNervous | 31.24 | 28.02 | 34.20 | 24.53 | 23.56 | 40.40 | 37.57 | 54.87 | 40.23 | 28.63 | 38.72 | 10.75 | 25.17 | 21.82 | 22.35 | 15.42 | 12.74 | 14.58 | 31.44 | 14.16 | 26.89 | 99.99 | 15.98 | 17.03 | 25.48 | 29.08 | 8.04 | NA | 0.14 | 0.10 |
| RecodeJittery | 20.88 | 24.05 | 34.31 | 31.92 | 30.18 | 52.84 | 42.66 | 38.79 | 36.63 | 26.40 | 39.84 | 18.27 | 15.29 | 30.88 | 18.22 | 23.72 | 37.49 | 21.12 | 31.98 | 18.33 | 26.59 | 19.75 | 30.14 | 20.75 | 16.98 | 29.38 | 18.99 | 31.95 | NA | -0.13 |
| RecodeScared | 46.71 | 45.51 | 61.24 | 51.16 | 41.33 | 55.29 | 46.83 | 64.14 | 71.98 | 30.93 | 27.03 | 23.62 | 22.20 | 34.41 | 26.17 | 36.16 | 17.94 | 17.03 | 35.43 | 12.14 | 24.21 | 99.94 | 24.05 | 35.82 | 23.88 | 75.84 | 21.11 | 18.84 | 30.65 | NA |

Note: For the local dependence (LD) type, the upper diagonal elements represent the standardized residuals in the form of signed Cramer's V Coefficient. SWLS01: "In most ways my life is close to my ideal"; SWLS02: "The conditions of my life are excellent"; SWLS03: "I am satisfied with my life"; SWLS04: "So far, I have gotten the important things I want in life"; and SWLS05: "If I could live my life over, I would change almost nothing"; HILS01: "My lifestyle allows me to be in harmony"; HILS02: "Most aspects of my life are in balance"; HILS03: "I am in harmony"; HILS04: "I accept the various conditions of my life"; and HILS05: "I fit in well with my surroundings".

Table S1b: The degrees of freedom and p-values for standardized residuals (Cramer's V Coefficient) among 30 items in all measures in the study (*n* = 435).

| Item | 1 | 2 | 3 | 4 | 5 | 6 | 7 | 8 | 9 | 10 | 11 | 12 | 13 | 14 | 15 | 16 | 17 | 18 | 19 | 20 | 21 | 22 | 23 | 24 | 25 | 26 | 27 | 28 | 29 | 30 |
|---|---|---|---|---|---|---|---|---|---|---|---|---|---|---|---|---|---|---|---|---|---|---|---|---|---|---|---|---|---|---|
| SWLS01 | NA | 0.00 | 0.00 | 0.00 | 0.00 | 0.00 | 0.026 | 0.032 | 0.00 | 0.008 | 0.002 | 0.19 | 0.00 | 0.026 | 0.00 | 0.00 | 0.00 | 0.00 | 0.003 | 0.046 | 0.387 | 0.084 | 0.001 | 0.137 | 0.013 | 0.373 | 0.22 | 0.147 | 0.646 | 0.004 |
| SWLS02 | 36.00 | NA | 0.00 | 0.00 | 0.00 | 0.184 | 0.00 | 0.002 | 0.00 | 0.004 | 0.006 | 0.533 | 0.012 | 0.118 | 0.029 | 0.298 | 0.089 | 0.03 | 0.017 | 0.382 | 0.00 | 0.002 | 0.007 | 0.035 | 0.004 | 0.272 | 0.164 | 0.259 | 0.459 | 0.005 |
| SWLS03 | 36.00 | 36.00 | NA | 0.00 | 0.00 | 0.00 | 0.00 | 0.00 | 0.001 | 0.01 | 0.035 | 0.14 | 0.00 | 0.014 | 0.041 | 0.079 | 0.012 | 0.004 | 0.004 | 0.033 | 0.127 | 0.00 | 0.002 | 0.053 | 0.042 | 0.088 | 0.005 | 0.081 | 0.079 | 0.00 |
| SWLS04 | 36.00 | 36.00 | 36.00 | NA | 0.00 | 0.00 | 0.00 | 0.001 | 0.00 | 0.018 | 0.132 | 0.251 | 0.012 | 0.071 | 0.043 | 0.042 | 0.06 | 0.00 | 0.00 | 0.034 | 0.00 | 0.00 | 0.061 | 0.133 | 0.005 | 0.056 | 0.208 | 0.432 | 0.129 | 0.001 |
| SWLS05 | 36.00 | 36.00 | 36.00 | 36.00 | NA | 0.00 | 0.00 | 0.00 | 0.00 | 0.017 | 0.001 | 0.154 | 0.002 | 0.303 | 0.01 | 0.016 | 0.042 | 0.389 | 0.019 | 0.032 | 0.359 | 0.161 | 0.014 | 0.169 | 0.079 | 0.117 | 0.066 | 0.487 | 0.179 | 0.015 |
| HILS01 | 36.00 | 36.00 | 36.00 | 36.00 | 36.00 | NA | 0.00 | 0.00 | 0.001 | 0.00 | 0.00 | 0.033 | 0.00 | 0.001 | 0.00 | 0.004 | 0.00 | 0.00 | 0.006 | 0.082 | 0.004 | 0.00 | 0.023 | 0.00 | 0.015 | 0.001 | 0.026 | 0.019 | 0.001 | 0.00 |
| HILS02 | 36.00 | 36.00 | 36.00 | 36.00 | 36.00 | 36.00 | NA | 0.00 | 0.001 | 0.001 | 0.004 | 0.039 | 0.00 | 0.002 | 0.00 | 0.007 | 0.00 | 0.00 | 0.00 | 0.012 | 0.00 | 0.011 | 0.028 | 0.00 | 0.02 | 0.001 | 0.052 | 0.038 | 0.011 | 0.004 |
| HILS03 | 36.00 | 36.00 | 36.00 | 36.00 | 36.00 | 36.00 | 36.00 | NA | 0.002 | 0.009 | 0.009 | 0.01 | 0.00 | 0.001 | 0.00 | 0.004 | 0.00 | 0.001 | 0.002 | 0.012 | 0.039 | 0.00 | 0.00 | 0.00 | 0.011 | 0.011 | 0.00 | 0.00 | 0.029 | 0.00 |
| HILS04 | 36.00 | 36.00 | 36.00 | 36.00 | 36.00 | 36.00 | 36.00 | 36.00 | NA | 0.00 | 0.161 | 0.01 | 0.03 | 0.077 | 0.587 | 0.193 | 0.004 | 0.012 | 0.022 | 0.237 | 0.001 | 0.025 | 0.005 | 0.004 | 0.129 | 0.013 | 0.224 | 0.02 | 0.048 | 0.00 |
| HILS05 | 36.00 | 36.00 | 36.00 | 36.00 | 36.00 | 36.00 | 36.00 | 36.00 | 36.00 | NA | 0.403 | 0.60 | 0.038 | 0.021 | 0.455 | 0.311 | 0.015 | 0.00 | 0.035 | 0.082 | 0.332 | 0.123 | 0.233 | 0.288 | 0.042 | 0.11 | 0.411 | 0.235 | 0.333 | 0.156 |
| Interested | 24.00 | 24.00 | 24.00 | 24.00 | 24.00 | 24.00 | 24.00 | 24.00 | 24.00 | 24.00 | NA | 0.486 | 0.00 | 0.00 | 0.27 | 0.004 | 0.001 | 0.004 | 0.027 | 0.536 | 0.002 | 0.062 | 0.037 | 0.209 | 0.268 | 0.029 | 0.025 | 0.001 | 0.001 | 0.041 |
| Enthusiastic | 24.00 | 24.00 | 24.00 | 24.00 | 24.00 | 24.00 | 24.00 | 24.00 | 24.00 | 24.00 | 16.00 | NA | 0.00 | 0.00 | 0.00 | 0.00 | 0.001 | 0.001 | 0.001 | 0.002 | 0.033 | 0.021 | 0.202 | 0.332 | 0.098 | 0.438 | 0.403 | 0.825 | 0.308 | 0.098 |
| Proud | 24.00 | 24.00 | 24.00 | 24.00 | 24.00 | 24.00 | 24.00 | 24.00 | 24.00 | 24.00 | 16.00 | 16.00 | NA | 0.00 | 0.02 | 0.007 | 0.00 | 0.00 | 0.00 | 0.001 | 0.002 | 0.003 | 0.081 | 0.056 | 0.106 | 0.279 | 0.482 | 0.067 | 0.504 | 0.137 |
| Alert | 24.00 | 24.00 | 24.00 | 24.00 | 24.00 | 24.00 | 24.00 | 24.00 | 24.00 | 24.00 | 16.00 | 16.00 | 16.00 | NA | 0.006 | 0.055 | 0.00 | 0.00 | 0.00 | 0.003 | 0.003 | 0.002 | 0.00 | 0.008 | 0.041 | 0.00 | 0.005 | 0.149 | 0.014 | 0.005 |
| Inspired | 24.00 | 24.00 | 24.00 | 24.00 | 24.00 | 24.00 | 24.00 | 24.00 | 24.00 | 24.00 | 16.00 | 16.00 | 16.00 | 16.00 | NA | 0.00 | 0.00 | 0.00 | 0.002 | 0.014 | 0.934 | 0.093 | 0.091 | 0.402 | 0.281 | 0.301 | 0.091 | 0.132 | 0.311 | 0.052 |
| Determined | 24.00 | 24.00 | 24.00 | 24.00 | 24.00 | 24.00 | 24.00 | 24.00 | 24.00 | 24.00 | 16.00 | 16.00 | 16.00 | 16.00 | 16.00 | NA | 0.00 | 0.006 | 0.002 | 0.00 | 0.053 | 0.154 | 0.005 | 0.356 | 0.33 | 0.02 | 0.382 | 0.494 | 0.096 | 0.003 |
| Attentive | 24.00 | 24.00 | 24.00 | 24.00 | 24.00 | 24.00 | 24.00 | 24.00 | 24.00 | 24.00 | 16.00 | 16.00 | 16.00 | 16.00 | 16.00 | 16.00 | NA | 0.00 | 0.00 | 0.148 | 0.111 | 0.137 | 0.001 | 0.139 | 0.032 | 0.00 | 0.001 | 0.692 | 0.002 | 0.327 |
| Active | 24.00 | 24.00 | 24.00 | 24.00 | 24.00 | 24.00 | 24.00 | 24.00 | 24.00 | 24.00 | 16.00 | 16.00 | 16.00 | 16.00 | 16.00 | 16.00 | 16.00 | NA | 0.013 | 0.038 | 0.485 | 0.471 | 0.401 | 0.214 | 0.231 | 0.373 | 0.257 | 0.556 | 0.174 | 0.383 |
| Excited | 24.00 | 24.00 | 24.00 | 24.00 | 24.00 | 24.00 | 24.00 | 24.00 | 24.00 | 24.00 | 16.00 | 16.00 | 16.00 | 16.00 | 16.00 | 16.00 | 16.00 | 16.00 | NA | 0.00 | 0.00 | 0.00 | 0.026 | 0.017 | 0.028 | 0.398 | 0.076 | 0.012 | 0.01 | 0.003 |
| Strong | 24.00 | 24.00 | 24.00 | 24.00 | 24.00 | 24.00 | 24.00 | 24.00 | 24.00 | 24.00 | 16.00 | 16.00 | 16.00 | 16.00 | 16.00 | 16.00 | 16.00 | 16.00 | 16.00 | NA | 0.03 | 0.039 | 0.164 | 0.143 | 0.84 | 0.834 | 0.83 | 0.587 | 0.305 | 0.734 |
| RecodedDistressed | 24.00 | 24.00 | 24.00 | 24.00 | 24.00 | 24.00 | 24.00 | 24.00 | 24.00 | 24.00 | 16.00 | 16.00 | 16.00 | 16.00 | 16.00 | 16.00 | 16.00 | 16.00 | 16.00 | 16.00 | NA | 0.00 | 0.401 | 0.001 | 0.112 | 0.2 | 0.05 | 0.043 | 0.046 | 0.085 |
| RecodedUpset | 24.00 | 24.00 | 24.00 | 24.00 | 24.00 | 24.00 | 24.00 | 24.00 | 24.00 | 24.00 | 16.00 | 16.00 | 16.00 | 16.00 | 16.00 | 16.00 | 16.00 | 16.00 | 16.00 | 16.00 | 16.00 | NA | 0.086 | 0.034 | 0.08 | 0.196 | 0.00 | 0.00 | 0.231 | 0.00 |
| RecodedGuilty | 24.00 | 24.00 | 24.00 | 24.00 | 24.00 | 24.00 | 24.00 | 24.00 | 24.00 | 24.00 | 16.00 | 16.00 | 16.00 | 16.00 | 16.00 | 16.00 | 16.00 | 16.00 | 16.00 | 16.00 | 16.00 | 16.00 | NA | 0.801 | 0.258 | 0.193 | 0.00 | 0.454 | 0.017 | 0.088 |
| RecodeAfraid | 24.00 | 24.00 | 24.00 | 24.00 | 24.00 | 24.00 | 24.00 | 24.00 | 24.00 | 24.00 | 16.00 | 16.00 | 16.00 | 16.00 | 16.00 | 16.00 | 16.00 | 16.00 | 16.00 | 16.00 | 16.00 | 16.00 | 16.00 | NA | 0.042 | 0.00 | 0.017 | 0.383 | 0.188 | 0.003 |
| RecodeHostile | 24.00 | 24.00 | 24.00 | 24.00 | 24.00 | 24.00 | 24.00 | 24.00 | 24.00 | 24.00 | 16.00 | 16.00 | 16.00 | 16.00 | 16.00 | 16.00 | 16.00 | 16.00 | 16.00 | 16.00 | 16.00 | 16.00 | 16.00 | 16.00 | NA | 0.011 | 0.322 | 0.062 | 0.387 | 0.092 |
| RecodeIrritable | 24.00 | 24.00 | 24.00 | 24.00 | 24.00 | 24.00 | 24.00 | 24.00 | 24.00 | 24.00 | 16.00 | 16.00 | 16.00 | 16.00 | 16.00 | 16.00 | 16.00 | 16.00 | 16.00 | 16.00 | 16.00 | 16.00 | 16.00 | 16.00 | 16.00 | NA | 0.002 | 0.023 | 0.022 | 0.00 |
| RecodeAshamed | 24.00 | 24.00 | 24.00 | 24.00 | 24.00 | 24.00 | 24.00 | 24.00 | 24.00 | 24.00 | 16.00 | 16.00 | 16.00 | 16.00 | 16.00 | 16.00 | 16.00 | 16.00 | 16.00 | 16.00 | 16.00 | 16.00 | 16.00 | 16.00 | 16.00 | 16.00 | NA | 0.948 | 0.269 | 0.174 |
| RecodeNervous | 24.00 | 24.00 | 24.00 | 24.00 | 24.00 | 24.00 | 24.00 | 24.00 | 24.00 | 24.00 | 16.00 | 16.00 | 16.00 | 16.00 | 16.00 | 16.00 | 16.00 | 16.00 | 16.00 | 16.00 | 16.00 | 16.00 | 16.00 | 16.00 | 16.00 | 16.00 | 16.00 | NA | 0.01 | 0.277 |
| RecodeJittery | 24.00 | 24.00 | 24.00 | 24.00 | 24.00 | 24.00 | 24.00 | 24.00 | 24.00 | 24.00 | 16.00 | 16.00 | 16.00 | 16.00 | 16.00 | 16.00 | 16.00 | 16.00 | 16.00 | 16.00 | 16.00 | 16.00 | 16.00 | 16.00 | 16.00 | 16.00 | 16.00 | 16.00 | NA | 0.015 |
| RecodeScared | 24.00 | 24.00 | 24.00 | 24.00 | 24.00 | 24.00 | 24.00 | 24.00 | 24.00 | 24.00 | 16.00 | 16.00 | 16.00 | 16.00 | 16.00 | 16.00 | 16.00 | 16.00 | 16.00 | 16.00 | 16.00 | 16.00 | 16.00 | 16.00 | 16.00 | 16.00 | 16.00 | 16.00 | 16.00 | NA |

Note: Lower diagonal = degrees of freedom. Upper diagonal = p-values. SWLS01: “In most ways my life is close to my ideal”; SWLS02: “The conditions of my life are excellent”; SWLS03: “I am satisfied with my life”; SWLS04: “So far, I have gotten the important things I want in life”; and SWLS05: “If I could live my life over, I would change almost nothing”; HILS01: “My lifestyle allows me to be in harmony”; HILS02: “Most aspects of my life are in balance”; HILS03: “I am in harmony”; HILS04: “I accept the various conditions of my life”; and HILS05: “I fit in well with my surroundings”.

Table S2: Q3 statistic to measures the local dependence violation among 30 items in all measures in the study. (*n* = 435).

| **Items** | 1 | 2 | 3 | 4 | 5 | 6 | 7 | 8 | 9 | 10 | 11 | 12 | 13 | 14 | 15 | 16 | 17 | 18 | 19 | 20 | 21 | 22 | 23 | 24 | 25 | 26 | 27 | 28 | 29 | 30 |
|---|---|---|---|---|---|---|---|---|---|---|---|---|---|---|---|---|---|---|---|---|---|---|---|---|---|---|---|---|---|---|
| **SWLS01** | 1.00 | 0.09 | 0.03 | 0.01 | -0.08 | 0.06 | 0.12 | 0.12 | 0.07 | -0.04 | 0.07 | -0.04 | 0.06 | -0.10 | -0.03 | -0.01 | -0.02 | -0.06 | 0.00 | -0.01 | 0.01 | 0.03 | -0.09 | 0.08 | 0.00 | -0.01 | -0.03 | 0.02 | -0.01 | 0.01 |
| **SWLS02** | 0.09 | 1.00 | -0.04 | -0.06 | -0.08 | 0.07 | 0.06 | 0.01 | 0.01 | -0.01 | -0.06 | 0.06 | 0.10 | -0.08 | 0.04 | 0.03 | 0.01 | 0.07 | 0.11 | 0.02 | 0.10 | 0.14 | -0.10 | -0.02 | -0.02 | 0.10 | -0.02 | 0.05 | -0.02 | -0.03 |
| **SWLS03** | 0.03 | -0.04 | 1.00 | **0.27** | 0.01 | 0.01 | 0.21 | 0.18 | **0.34** | 0.12 | 0.09 | 0.13 | 0.09 | -0.08 | -0.10 | 0.00 | 0.00 | -0.03 | -0.08 | 0.10 | 0.04 | 0.03 | -0.06 | 0.02 | 0.05 | -0.01 | -0.05 | -0.02 | -0.01 | -0.03 |
| **SWLS04** | 0.01 | -0.06 | 0.27 | 1.00 | 0.07 | 0.03 | 0.07 | 0.02 | 0.10 | 0.10 | 0.02 | 0.07 | -0.01 | 0.06 | -0.10 | -0.04 | 0.04 | 0.00 | -0.06 | 0.00 | 0.04 | 0.04 | -0.10 | 0.01 | 0.07 | 0.04 | -0.06 | 0.05 | 0.04 | 0.04 |
| **SWLS05** | -0.08 | -0.08 | 0.01 | 0.07 | 1.00 | -0.02 | 0.02 | 0.02 | -0.01 | 0.00 | -0.15 | 0.01 | 0.04 | 0.03 | 0.11 | 0.03 | 0.01 | 0.01 | 0.08 | 0.01 | -0.03 | 0.01 | 0.01 | 0.01 | -0.07 | -0.03 | -0.04 | -0.07 | -0.06 | -0.07 |
| **HILS01** | 0.06 | 0.07 | 0.01 | 0.03 | -0.02 | 1.00 | 0.06 | 0.04 | -0.05 | -0.13 | 0.03 | 0.04 | 0.03 | -0.05 | -0.01 | 0.00 | -0.01 | 0.02 | -0.02 | 0.06 | -0.02 | -0.04 | -0.07 | -0.10 | -0.03 | 0.01 | -0.05 | 0.00 | -0.02 | -0.12 |
| **HILS02** | 0.12 | 0.06 | 0.21 | 0.07 | 0.02 | 0.06 | 1.00 | 0.08 | 0.07 | 0.02 | 0.09 | 0.05 | 0.13 | -0.08 | -0.06 | -0.02 | 0.03 | 0.04 | 0.04 | 0.03 | 0.13 | 0.08 | 0.06 | 0.10 | 0.05 | 0.04 | 0.08 | 0.08 | 0.05 | -0.02 |
| **HILS03** | 0.12 | 0.01 | 0.18 | 0.02 | 0.02 | 0.04 | 0.08 | 1.00 | 0.10 | -0.02 | 0.07 | -0.03 | 0.04 | -0.09 | 0.10 | -0.02 | -0.05 | 0.01 | 0.01 | 0.04 | 0.21 | 0.08 | -0.01 | -0.02 | -0.01 | 0.09 | -0.03 | 0.01 | 0.07 | -0.05 |
| **HILS04** | 0.07 | 0.01 | 0.34 | 0.10 | -0.01 | -0.05 | 0.07 | 0.10 | 1.00 | 0.11 | 0.00 | 0.05 | 0.06 | 0.00 | -0.01 | 0.04 | 0.14 | 0.05 | -0.09 | 0.04 | -0.02 | 0.05 | -0.01 | 0.05 | 0.11 | 0.11 | -0.04 | -0.01 | -0.05 | 0.02 |
| **HILS05** | -0.04 | -0.01 | 0.12 | 0.10 | 0.00 | -0.13 | 0.02 | -0.02 | 0.11 | 1.00 | 0.05 | 0.01 | 0.10 | -0.02 | 0.05 | -0.06 | 0.00 | 0.03 | 0.04 | 0.00 | 0.06 | 0.01 | -0.13 | 0.06 | 0.03 | 0.00 | -0.01 | 0.04 | 0.04 | 0.13 |
| **Interested** | 0.07 | -0.06 | 0.09 | 0.02 | -0.15 | 0.03 | 0.09 | 0.07 | 0.00 | 0.05 | 1.00 | 0.04 | -0.06 | -0.01 | -0.13 | -0.04 | 0.02 | 0.04 | -0.18 | -0.01 | 0.03 | -0.02 | -0.01 | 0.01 | -0.09 | -0.07 | 0.06 | 0.08 | 0.03 | 0.03 |
| **Enthusiastic** | -0.04 | 0.06 | 0.13 | 0.07 | 0.01 | 0.04 | 0.05 | -0.03 | 0.05 | 0.01 | 0.04 | 1.00 | 0.08 | -0.08 | 0.08 | -0.12 | 0.02 | 0.05 | **0.24** | -0.02 | 0.02 | 0.09 | -0.05 | -0.11 | -0.08 | 0.13 | -0.07 | -0.03 | -0.08 | -0.08 |
| **Proud** | 0.06 | 0.10 | 0.09 | -0.01 | 0.04 | 0.03 | 0.13 | 0.04 | 0.06 | 0.10 | -0.06 | 0.08 | 1.00 | -0.07 | 0.21 | -0.04 | -0.15 | -0.02 | 0.14 | 0.21 | 0.07 | 0.06 | -0.06 | 0.10 | -0.05 | 0.00 | 0.03 | 0.10 | 0.01 | 0.05 |
| **Alert** | -0.10 | -0.08 | -0.08 | 0.06 | 0.03 | -0.05 | -0.08 | -0.09 | 0.00 | -0.02 | -0.01 | -0.08 | -0.07 | 1.00 | -0.04 | 0.06 | **0.33** | -0.05 | -0.15 | -0.07 | -0.08 | -0.14 | -0.01 | -0.05 | -0.01 | -0.08 | 0.00 | -0.07 | -0.04 | 0.03 |
| **Inspired** | -0.03 | 0.04 | -0.10 | -0.10 | 0.11 | -0.01 | -0.06 | 0.10 | -0.01 | 0.05 | -0.13 | 0.08 | 0.21 | -0.04 | 1.00 | 0.02 | -0.16 | 0.05 | **0.27** | 0.17 | 0.01 | 0.04 | -0.01 | 0.06 | -0.01 | 0.08 | -0.04 | 0.06 | -0.08 | -0.02 |
| **Determined** | -0.01 | 0.03 | 0.00 | -0.04 | 0.03 | 0.00 | -0.02 | -0.02 | 0.04 | -0.06 | -0.04 | -0.12 | -0.04 | 0.06 | 0.02 | 1.00 | 0.19 | -0.10 | -0.06 | 0.11 | -0.11 | 0.01 | 0.15 | 0.02 | 0.09 | -0.06 | 0.03 | 0.01 | 0.06 | -0.02 |
| **Attentive** | -0.02 | 0.01 | 0.00 | 0.04 | 0.01 | -0.01 | 0.03 | -0.05 | 0.14 | 0.00 | 0.02 | 0.02 | -0.15 | 0.33 | -0.16 | 0.19 | 1.00 | 0.04 | -0.13 | -0.09 | -0.04 | 0.05 | 0.07 | -0.02 | 0.10 | 0.05 | 0.07 | -0.15 | 0.00 | -0.02 |
| **Active** | -0.06 | 0.07 | -0.03 | 0.00 | 0.01 | 0.02 | 0.04 | 0.01 | 0.05 | 0.03 | 0.04 | 0.05 | -0.02 | -0.05 | 0.05 | -0.10 | 0.04 | 1.00 | 0.08 | 0.09 | 0.07 | 0.07 | -0.10 | -0.03 | -0.06 | 0.04 | -0.01 | 0.01 | -0.04 | 0.01 |
| **Excited** | 0.00 | 0.11 | -0.08 | -0.06 | 0.08 | -0.02 | 0.04 | 0.01 | -0.09 | 0.04 | -0.18 | 0.24 | 0.14 | -0.15 | 0.27 | -0.06 | -0.13 | 0.08 | 1.00 | 0.09 | 0.00 | 0.05 | -0.11 | -0.08 | -0.11 | 0.01 | -0.05 | -0.04 | -0.15 | -0.03 |
| **Strong** | -0.01 | 0.02 | 0.10 | 0.00 | 0.01 | 0.06 | 0.03 | 0.04 | 0.04 | 0.00 | -0.01 | -0.02 | 0.21 | -0.07 | 0.17 | 0.11 | -0.09 | 0.09 | 0.09 | 1.00 | 0.01 | 0.09 | -0.04 | 0.01 | 0.00 | 0.02 | -0.01 | 0.07 | 0.06 | 0.00 |
| **RecodedDistressed** | 0.01 | 0.10 | 0.04 | 0.04 | -0.03 | -0.02 | 0.13 | 0.21 | -0.02 | 0.06 | 0.03 | 0.02 | 0.07 | -0.08 | 0.01 | -0.11 | -0.04 | 0.07 | 0.00 | 0.01 | 1.00 | **0.48** | 0.02 | 0.14 | 0.05 | **0.28** | 0.11 | 0.15 | 0.08 | **0.26** |
| **RecodedUpset** | 0.03 | 0.14 | 0.03 | 0.04 | 0.01 | -0.04 | 0.08 | 0.08 | 0.05 | 0.01 | -0.02 | 0.09 | 0.06 | -0.14 | 0.04 | 0.01 | 0.05 | 0.07 | 0.05 | 0.09 | 0.48 | 1.00 | 0.16 | 0.21 | 0.14 | **0.32** | 0.06 | 0.02 | 0.04 | 0.16 |
| **RecodedGuilty** | -0.09 | -0.10 | -0.06 | -0.10 | 0.01 | -0.07 | 0.06 | -0.01 | -0.01 | -0.13 | -0.01 | -0.05 | -0.06 | -0.01 | -0.01 | 0.15 | 0.07 | -0.10 | -0.11 | -0.04 | 0.02 | 0.16 | 1.00 | **0.29** | 0.19 | 0.07 | **0.49** | 0.08 | 0.10 | 0.15 |
| **RecodeAfraid** | 0.08 | -0.02 | 0.02 | 0.01 | 0.01 | -0.10 | 0.10 | -0.02 | 0.05 | 0.06 | 0.01 | -0.11 | 0.10 | -0.05 | 0.06 | 0.02 | -0.02 | -0.03 | -0.08 | 0.01 | 0.14 | 0.21 | 0.29 | 1.00 | 0.17 | -0.03 | 0.22 | 0.22 | 0.16 | **0.56** |
| **RecodeHostile** | 0.00 | -0.02 | 0.05 | 0.07 | -0.07 | -0.03 | 0.05 | -0.01 | 0.11 | 0.03 | -0.09 | -0.08 | -0.05 | -0.01 | -0.01 | 0.09 | 0.10 | -0.06 | -0.11 | 0.00 | 0.05 | 0.14 | 0.19 | 0.17 | 1.00 | **0.34** | 0.19 | 0.07 | 0.16 | 0.16 |
| **RecodeIrritable** | -0.01 | 0.10 | -0.01 | 0.04 | -0.03 | 0.01 | 0.04 | 0.09 | 0.11 | 0.00 | -0.07 | 0.13 | 0.00 | -0.08 | 0.08 | -0.06 | 0.05 | 0.04 | 0.01 | 0.02 | 0.28 | 0.32 | 0.07 | -0.03 | 0.34 | 1.00 | 0.06 | 0.12 | 0.20 | -0.01 |
| **RecodeAshamed** | -0.03 | -0.02 | -0.05 | -0.06 | -0.04 | -0.05 | 0.08 | -0.03 | -0.04 | -0.01 | 0.06 | -0.07 | 0.03 | 0.00 | -0.04 | 0.03 | 0.07 | -0.01 | -0.05 | -0.01 | 0.11 | 0.06 | 0.49 | 0.22 | 0.19 | 0.06 | 1.00 | 0.20 | 0.11 | **0.28** |
| **RecodeNervous** | 0.02 | 0.05 | -0.02 | 0.05 | -0.07 | 0.00 | 0.08 | 0.01 | -0.01 | 0.04 | 0.08 | -0.03 | 0.10 | -0.07 | 0.06 | 0.01 | -0.15 | 0.01 | -0.04 | 0.07 | 0.15 | 0.02 | 0.08 | 0.22 | 0.07 | 0.12 | 0.20 | 1.00 | **0.37** | **0.29** |
| **RecodeJittery** | -0.01 | -0.02 | -0.01 | 0.04 | -0.06 | -0.02 | 0.05 | 0.07 | -0.05 | 0.04 | 0.03 | -0.08 | 0.01 | -0.04 | -0.08 | 0.06 | 0.00 | -0.04 | -0.15 | 0.06 | 0.08 | 0.04 | 0.10 | 0.16 | 0.16 | 0.20 | 0.11 | 0.37 | 1.00 | 0.22 |
| **RecodeScared** | 0.01 | -0.03 | -0.03 | 0.04 | -0.07 | -0.12 | -0.02 | -0.05 | 0.02 | 0.13 | 0.03 | -0.08 | 0.05 | 0.03 | -0.02 | -0.02 | -0.02 | 0.01 | -0.03 | 0.00 | 0.26 | 0.16 | 0.15 | 0.56 | 0.16 | -0.01 | 0.28 | 0.29 | 0.22 | 1.00 |

Note: bold type is more than absolute value (0.2236) as suggested by Yen (1984) that indicates to the violation against local independence. SWLS01: "In most ways my life is close to my ideal"; SWLS02: "The conditions of my life are excellent"; SWLS03: "I am satisfied with my life"; SWLS04: "So far, I have gotten the important things I want in life"; and SWLS05: "If I could live my life over, I would change almost nothing"; HILS01: "My lifestyle allows me to be in harmony"; HILS02: "Most aspects of my life are in balance"; HILS03: "I am in harmony"; HILS04: "I accept the various conditions of my life"; and HILS05: "I fit in well with my surroundings".

Table S3: Indices of item fit and Benjamini-Hochberg criterion for p-value adjustment among 30 items in all measures in the study (*n* = 435).

| **Satisfaction with Life Scale (SWLS)** | **S_X2** | **df.S_X2** | **RMSEA.S_X2** | **P.S_X2** | **Rank** | **(i/m)*Q** |
|---|---|---|---|---|---|---|
| SWLS01 | 202.502 | 63.00 | 0.071 | 0.00 | 1 | 0.00 |
| SWLS02 | 159.67 | 85.00 | 0.05 | 0.00 | 2 | 0.00 |
| SWLS03 | 169.44 | 75.00 | 0.05 | 0.00 | 3 | 0.01 |
| SWLS04 | 105.05 | 92.00 | 0.02 | 0.17 | 5 | 0.01 |
| SWLS05 | 156.43 | 129.00 | 0.02 | 0.05 | 4 | 0.01 |
| **Harmony in Life Scale (HILS)** | | | | | | |
| HILS01 | 151.39 | 60.00 | 0.06 | 0.00 | 1 | 0.00 |
| HILS02 | 150.38 | 60.00 | 0.06 | 0.00 | 2 | 0.00 |
| HILS03 | 151.88 | 63.00 | 0.06 | 0.00 | 3 | 0.01 |
| HILS04 | 161.57 | 69.00 | 0.06 | 0.00 | 4 | 0.01 |
| HILS05 | 102.30 | 77.00 | 0.03 | 0.03 | 5 | 0.01 |
| **Positive Affect (PA)** | | | | | | |
| Interested | 110.00 | 63.00 | 0.04 | 0.00 | 1 | 0.00 |
| Enthustiastic | 101.10 | 83.00 | 0.02 | 0.09 | 9 | 0.01 |
| Proud | 113.69 | 93.00 | 0.02 | 0.07 | 7 | 0.01 |
| Alert | 137.62 | 84.00 | 0.04 | 0.00 | 2 | 0.00 |
| Inspired | 118.60 | 97.00 | 0.02 | 0.07 | 8 | 0.01 |
| Determined | 97.67 | 70.00 | 0.03 | 0.02 | 5 | 0.01 |
| Attentive | 90.61 | 71.00 | 0.03 | 0.06 | 6 | 0.01 |
| Active | 87.37 | 71.00 | 0.02 | 0.09 | 10 | 0.01 |
| Excited | 133.16 | 95.00 | 0.03 | 0.01 | 3 | 0.00 |
| Strong | 124.10 | 90.00 | 0.03 | 0.01 | 4 | 0.00 |
| **Negative Affect (NA)** | | | | | | |
| RecodedDistressed | 151.07 | 69.00 | 0.05 | 0.00 | 1 | 0.00 |
| RecodedUpset | 138.76 | 72.00 | 0.05 | 0.00 | 2 | 0.00 |
| RecodedGuilty | 145.58 | 64.00 | 0.05 | 0.00 | 3 | 0.00 |
| RecodeAfraid | 132.54 | 65.00 | 0.05 | 0.00 | 4 | 0.00 |
| RecodeHostile | 84.76 | 67.00 | 0.03 | 0.07 | 10 | 0.01 |
| RecodeIrritable | 118.81 | 87.00 | 0.03 | 0.01 | 9 | 0.01 |
| RecodeAshamed | 112.27 | 57.00 | 0.05 | 0.00 | 5 | 0.01 |
| RecodeNervous | 151.70 | 83.00 | 0.04 | 0.00 | 6 | 0.01 |
| RecodeJittery | 112.13 | 74.00 | 0.03 | 0.00 | 7 | 0.01 |
| RecodeScared | 156.36 | 63.00 | 0.06 | 0.00 | 8 | 0.01 |

Note: i = individual p-value's rank, m = total number of tests, Q = false discovery rate = .01. SWLS01: "In most ways my life is close to my ideal"; SWLS02: "The conditions of my life are excellent"; SWLS03: "I am satisfied with my life"; SWLS04: "So far, I have gotten the important things I want in life"; and SWLS05: "If I could live my life over, I would change almost nothing"; HILS01: "My lifestyle allows me to be in harmony"; HILS02: "Most aspects of my life are in balance"; HILS03: "I am in harmony"; HILS04: "I accept the various conditions of my life"; and HILS05: "I fit in well with my surroundings".
